# Characterizing Hox genes in mayflies (Ephemeroptera), with *Hexagenia limbata* as a new mayfly model

**DOI:** 10.1186/s13227-022-00200-w

**Published:** 2022-07-27

**Authors:** Christopher J. Gonzalez, Tobias R. Hildebrandt, Brigid O’Donnell

**Affiliations:** 1 Biological Sciences, Plymouth State University, Plymouth, NH USA; 2 Computational and Applied Mathematic Science, Plymouth State University, Plymouth, NH USA

**Keywords:** Body plan evolution, Ephemeroptera, Model organism, Mayfly gills, Hox genes, Transcriptome, Immunohistochemistry

## Abstract

**Background:**

Hox genes are key regulators of appendage development in the insect body plan. The body plan of mayfly (Ephemeroptera) nymphs differs due to the presence of abdominal appendages called gills. Despite mayflies’ phylogenetic position in Paleoptera and novel morphology amongst insects, little is known of their developmental genetics, such as the appendage-regulating Hox genes. To address this issue we present an annotated, early instar transcriptome and embryonic expression profiles for Antennapedia, Ultrabithorax, and Abdominal A proteins in the mayfly *Hexagenia limbata,* identify putative Hox protein sequences in the mayflies *H. limbata*, *Cloeon dipterum*, and *Ephemera danica*, and describe the genomic organization of the Hox gene cluster in *E. danica*.

**Results:**

Transcriptomic sequencing of early instar *H. limbata* nymphs yielded a high-quality assembly of 83,795 contigs, of which 22,975 were annotated against *Folsomia candida*, *Nilaparvata lugens*, *Zootermopsis nevadensis* and UniRef90 protein databases. Homeodomain protein phylogeny and peptide annotations identified coding sequences for eight of the ten canonical Hox genes (excluding *zerknüllt/Hox3* and *fushi tarazu*) in *H. limbata* and *C. dipterum*, and all ten in *E. danica*. Mayfly Hox protein sequences and embryonic expression patterns of Antp, Ubx, and Abd-A appear highly conserved with those seen in other non-holometabolan insects. Similarly, the genomic organization of the Hox cluster in *E. danica* resembles that seen in most insects.

**Conclusions:**

We present evidence that mayfly Hox peptide sequences and the embryonic expression patterns for Antp, Ubx, and Abd-A are extensively conserved with other insects, as is organization of the mayfly Hox gene cluster. The protein data suggest mayfly Antp, Ubx, and Abd-A play appendage promoting and repressing roles during embryogenesis in the thorax and abdomen, respectively, as in other insects. The identified expression of eight Hox genes, including *Ubx* and *abd-A*, in early instar nymphs further indicates a post-embryonic role, possibly in gill development. These data provide a basis for *H. limbata* as a complementary Ephemeridae model to the growing repertoire of mayfly model species and molecular techniques.

**Supplementary Information:**

The online version contains supplementary material available at 10.1186/s13227-022-00200-w.

## Background

Arthropods are the most speciose clade of animals on earth, an evolutionary success widely attributed to the evolution and diversification of segmented body plans [1]. Of particular note is the insect body plan, which consists of a head with antennae and gnathal appendages, a thorax with three pairs of walking legs, and an abdomen largely devoid of appendages except external genitalia [2, 3]. This relatively simple body plan is the basis for a vast range of appendage diversification. One example is the transition of ancestral gnathal appendages to piercing and sucking structures in hemipterans, an elongated proboscis in many lepidopterans, a sponge-like proboscis in many dipterans, and structures adapted for nest construction or defense, as in numerous hymenopterans [4, 5]. Similarly, the thoracic leg segments may be elongated for mobility on the water surface, as in some hemipterans, or enlarged for jumping, as in many orthopterans. With such immense diversity, insects provide unique opportunities to study the evolutionary mechanisms of body patterning and appendage diversification.

From the perspective of developmental genetics, insect appendage diversity has been explained in part by the Hox genes, a highly conserved gene family first characterized in the fruit fly *Drosophila melanogaster* [6, 7]. The canonical Hox family comprises ten genes organized on a single chromosome, each expressed along the anterior–posterior axis of the embryo in parallel with their chromosomal order [8–11]. Hox genes are key factors in regulating body patterning and appendage identity, developing unique appendage phenotypes, and shifting appendage morphology [11–17]. The genomic organization of Hox genes varies [18], but in *Drosophila* is comprised of two complexes. The anterior Antennapedia complex is key for specifying the development of antennae, gnathal appendages, and thoracic legs, and consists of the genes *labial (lab)*, *proboscipedia (pb)*, *zerknüllt*/*Hox3 (zen), Deformed (Dfd)*, *Sex combs reduced (Scr)*, *fushi tarazu* (*ftz*) and *Antennapedia (Antp)* [11]. The posterior Bithorax complex plays a central role in specifying the largely appendage-less abdomen, and contains the genes *Ultrabithorax (Ubx)*, *abdominal A (abd-A)*, and *Abdominal B (Abd-B)* [11]. All Hox proteins contain a DNA binding homeodomain, and can be further distinguished by the presence or absence of several conserved functional regions, such as the SSYF motif, hexapeptide, and UbdA motif [19, 20]. Collectively, changes in Hox gene expression and function are key for the evolution of novelties in the insect body plan, such as specialized thoracic legs in some orthopteran and hemipteran species, and possibly the abdominal appendages of ephemeropterans [21–23].

Mayflies (Ephemeroptera) belong to one of the earliest branching clades of winged insects [24, 25], and mature from aquatic nymphs that develop paired abdominal gills on the first seven abdominal segments (tional file 1: Fig. S1). Gill morphology is incredibly diverse, ranging from small thin threads to flattened, leaf-like lamellae, highly sclerotized plates, and bilamellate, feathery structures. Moreover, gill position on the abdomen may also be dorsal, lateral, or ventral, and many species exhibit shape and size differences in gills along the abdominal segments [26, 27]. Coincident with this morphological diversity, gills serve a variety of functions in food acquisition/water movement, locomotion, oxygen and ion uptake, protection of other gills, and adherence to the substrate (e.g.*, *[28–30]). The unique morphology and functional roles of mayfly gills represent a distinct divergence from the appendage-less abdomen that defines most insects, raising key questions on the origins of the insect body plan and the evolution of novel appendage types, such as wings.

The evolutionary position of mayflies has sparked recent interest in their genetics. The first mayfly transcriptome for *Cloeon viridulum* (Baetidae) was sequenced to study differential gene expression during metamorphosis, and a genome and several transcriptomes of *C. dipterum* were sequenced to assess mayfly lifecycle adaptations and support the development of *C. dipterum* as an emerging model system [31–33]. However, homolog annotations for mayfly transcriptomic and genomic resources remain absent; next-generation sequencing outside the Baetidae family is also sparse, as is the availability of data for Hox genes from early clades of winged insects. Furthermore, only four studies have documented protocols for gene expression patterns during mayfly development, with three focusing on embryonic expression patterns, and one on expression within the gills and heads of nymphs [32–35]. Readily applicable genetic resources for mayfly developmental studies are thus limited, and the genomic organization, full protein sequence, and expression patterns of mayfly Hox genes remains unknown.

To address these limitations, we assembled an annotated transcriptome for early instar nymphs of the burrowing mayfly *Hexagenia limbata* (Ephemeridae [36]), and annotated putative Hox protein sequences for the mayflies *H. limbata*, *Ephemera danica* (Ephemeridae), and *C. dipterum* (Baetidae). We further examined the organization of the Hox gene cluster in *E. danica*, and provide the first report of spatial Hox gene expression data in a mayfly, focusing on *H. limbata* Antp, Ubx, and Abd-A from early to late embryogenesis. Finally, we assess the current status of mayflies as a model in evo devo, and what technical challenges remain.

## Methods

### Egg collection and maintenance

Mature *H. limbata* females were collected by black lighting at Sky Pond (New Hampton, Belknap Co., NH) on peak hatch nights in June and July, 2013–2016. Eggs were extracted from captured females by submerging the abdomen into a conical tube containing pond water to stimulate egg laying [36]. Egg production amongst female mayflies tends to scale with overall body size, with each large female *Hexagenia* producing ~ 8000 eggs [37–39].

Collected eggs were washed in a solution of 10% bleach and rinsed thoroughly with aged (24 h) distilled water. All eggs were maintained in aged distilled water at room temperature (approximately 25 °C) until fixation or reaching approximately 50% development, then stored at 4 °C to induce a diapause-like state [40] for future study. Hatched nymphs were reared in glass containers with aged distilled water, while older nymphs were collected directly from pond mud samples and housed in containers filled with pond water and mud. All nymphs were maintained at room temperature and ambient light conditions (approximately 12-h light–dark cycles).

### Immunohistochemistry

Embryos were fixed by modifying an established protocol [35]. Live eggs were first washed thoroughly with PBTw (1X phosphate-buffered saline + 0.1% Tween), soaked for six minutes in a 50% bleach solution to remove the chorion, then fixed for 30–50 min with agitation in a 6% formaldehyde and PBTw fixative with heptanes at a 2:1 ratio. Following fixation, eggs were washed in PBTw and stored at − 20 °C in absolute methanol.

Fixed eggs were rinsed in PBTw and stripped of the vitelline membrane by submersion in a waterbath sonicator (Fisher Scientific FS20D) at 42 kHz (± 6%) for several seconds. Embryos were then soaked in SuperBlock T20 (Thermo Scientific, MA) for 30 min at room temperature and incubated overnight at 4 °C in either 12 ng/µl of Antp 4C3 (DSHB, University of Iowa; deposited by Brower, D.) or 20 ng/µl of Ubx/Abd-A FP6.87 primary antibody (DSHB, University of Iowa; deposited by White, R.) diluted in SuperBlock T20. Following primary antibody incubation, the embryos were washed with PBTw for 1 h (1 wash/10 min), then incubated for 2 h in a 1:500 dilution of horseradish–peroxidase conjugated goat anti-mouse secondary antibody (Jackson ImmunoResearch, PA) in SuperBlock T20. Embryos were washed again as above in PBTw, equilibrated for twenty min (1 wash/5 min) in 1X stable peroxide buffer (1XHP) (Thermo Scientific, MA), and developed for ten minutes using 1:10 dilution of metal-enhanced diaminobenzidine substrate (Thermo Scientific, MA) in 1XHP buffer. After developing, embryos were washed in PBTw, counterstained with a 1:1000 dilution (1 µg/mL) of DAPI (Pierce Biotechnology), and stored at − 20 °C in 80% glycerol. Negative control embryos of all stages were incubated in SuperBlock T20 instead of primary antibody and showed little sign of non-specific staining (Additional file 1: Fig. S2).

Embryos were imaged on a BX53 Olympus compound microscope using differential interference contrast optics, a Q-Color 5 Olympus camera, and QCapture Suite Plus v.3.1.3.10 (QImaging, Surrey, BC, Canada). Nymphs were imaged on a Leica EZ4 HD stereomicroscope with Leica Aquire v.1.0 (Buffalo Grove, IL, USA). Image values for exposure, contrast, light balance, and color were adjusted in Keynote v.6.6.2 to improve quality (Additional file 1: Fig. S2). Scale bars for all images were calibrated in Image J v.1.46r [41]. DAPI-based pencil sketches overlaid on the images to clarify morphology were drawn in Gimp v2.10.14.

### Nymphal cDNA library preparation

We used approximately 100 μl of whole-body nymphs as starting material to represent the full set of expressed genes present in early nymphal development. Of the nymphs used for RNA sequencing, most were first instar, and the rest second instar. Total mRNA was extracted using TRIzol (Ambion), then column purified with RNeasy (Qiagen). Purified mRNA was treated with Turbo DNase (Ambion), quantified, and checked for purity with a NanoDrop 2000 (Wilmington, DE) before storage at -80℃.

Purified mRNA was sent to the Hubbard Center for Genome Studies (University of New Hampshire, Durham, NH) and checked for quality and quantity with an Agilent 2100 Bioanalyzer (Agilent Technologies, Santa Clara, CA). An Illumina compatible library was constructed using an Illumina TruSeq RNA Prep Kit V2 with index Set A (RS-122-2101), following the low sample input protocol (Part #15,026,495 Rev. F). Briefly, 1 μg of total mRNA was used as initial input; mRNA was then purified, fragmented, and primed with random hexamers using poly-T oligo attached magnetic beads. cDNA was reverse-transcribed with SuperScript II Reverse Transcriptase, 3’ adenylated, ligated with RNA adapter indices, and PCR-enriched. Finally, the cDNA library was checked for quality with an Agilent 2100 Bioanalyzer and normalized to 10 nM prior to sequencing.

### Transcriptome assembly & assessment

Raw paired-end reads were quality-checked using FastQC (v0.11.9; https://www.bioinformatics.babraham.ac.uk/projects/fastqc/) before transcriptomic assembly. Two assemblies were then made, first via the de novo assembly option in CLC Genomics WorkBench v.6.0.4 (CLCBio, Boston, MA) using raw reads, with scaffolding enabled, a minimum contig length of 200 bp, and automatic word and bubble sizes of 24 and 50, respectively. The second was assembled using the Oyster River Protocol (ORP, v2.2.6; [42]) and default settings (TPM_FILT = 1, STRAND = RF, MEM = 150, CPU = 24), on an Amazon Web Service EC2 server with 32 vCPUs and 128 GB of RAM. The first step in the ORP pipeline is read error correction via Rcorrector (v1.0.3), followed by Illumina adaptor removal and trimming of reads with Phred quality below 3, using Trimmomatic (v0.38). Trinity (v2.8.4; [43]), Spades55, Spades75 (v3.13.0; [44]) and Transabyss (v2.0.1; [45]) are then used to make four de novo assemblies, which are merged via a modified version of Orthofinder (v2.2.6; [46]) packaged in OrthoFuser [45]. The CLC and ORP-merged assemblies were assessed via BUSCO (v4.0.6; [47]) with the insecta_odb10 database, and TransRate (v1.0.3; [48]) read mapping.

### Transcriptome annotation

Full transcriptome annotation was done using Diamond (version 0.9.24.125; [49]) on an Amazon Web Service EC2 server (48 vCPUs, 192 GB of RAM, and one 900 NVMe SSD), with the default *e* value cutoff of 0.001 against a merged protein database containing sequences from *Folsomia candida* (springtail), *Nilaparvata lugens* (brown planthopper), *Zootermopsis nevadensis* (termite) and UniRef90 (https://www.uniprot.org/downloads; [50]). Hox-specific annotations were further supported via a reciprocal blast pipeline, in which *D. melanogaster* protein homologs for Lab, Pb, Zen, Dfd, Scr, Ftz, Antp, Ubx, Abd-A, and Abd-B were aligned against ORP contigs with tBLASTn [51]. Because the Zen and Ftz sequences differ notably from many insects in *Drosophila* and have been difficult to identify in early branching insects, additional blasts with Zen and Ftz homologs for the springtail *Folsomia candida*, brown planthopper *Nilaparvata lugens*, thrip *Frankliniella occidentalis*, locust *Schistocerca gregaria*, and red flour beetle *Tribolium castaneum* were conducted. All contigs from blast outputs with an *e* value of 1e-20 or less were translated with TransDecoder (v5.5.0; https://github.com/TransDecoder/TransDecoder/wiki), then reciprocally blasted via BLASTp against the NCBI non-redundant protein database (http://blast.ncbi.nlm.nih.gov/). To further explore mayfly Hox sequences, the reciprocal blast pipeline was then used with genome-based protein data sets for the mayflies *E. danica* and *C. dipterum*. All mayfly proteins with consistent hits for a given Hox gene were aligned to insect Hox homologs with the MAFFT L-INS-i algorithm (v7.471; [52, 53]) and annotated for functional domains using Genomic SMART (v8.0; [54]). All accession values for publicly available sequences are provided in Additional file 1: Table S1.

### Hox peptide phylogeny

Genome-derived peptide data sets were downloaded from NCBI for fifteen hexapod species, including *Orchesella cincta, Folsomia candida, Ephemera danica, Cloeon dipterum, Ladona fulva, Blattella germanica, Zootermopsis nevadensis, Diuraphis noxia, Thrips palmi, Frankliniella occidentalis, Apis mellifera, Tribolium castaneum, Chrysoperla carnea, Bombyx mori,* and *Drosophila melanogaster* (See Additional file 1: Table S1 for genome accession numbers). All genomic assemblies met a minimum coverage of 7X for Sanger data [18] and at least 40X for Illumina or PacBio methods. For *Hexagenia limbata*, a peptide data set (Additional file 1: Table S1) was generated from the ORP assembly via Transdecoder (v3.0.1). Phylogenetic Focusing (v2.0; https://github.com/C-gonz/Phylogenetic_Focusing) was used with these protein data sets to generate a homeobox gene phylogeny. In brief, a fasta file of Hox, ANTP-class, and non ANTP-class homeodomain protein homologs from *D. melanogaster* and non-holometabolan insects (see Additional file 1: Table S1 for query peptides and accession numbers) was queried using BLASTp against each species’ peptide data set, with the default *e* value cutoff of 1e-05. For each species, all hit sequences are run through CD-HIT (v4.7; *c* = 0.98, *n* = 5; [55, 56]) to reduce duplicate sequences, aligned with MAFFT (v7.305b), and run through IQ-Tree (v1.6.12; [57]) with default parameters (m = MFP + C60, bb = 1000, -alrt = 1000, -nt = 24) to produce a gene phylogeny. Each species’ gene phylogeny was then rooted with the non ANTP-class clade, and the subclade containing ANTP-class homeodomain proteins was extracted. Extracted subtree sequences were concatenated, filtered to remove duplicates and non-homeodomain proteins via CD-HIT and HMMR (v.3.2.1 [58], using a MAFFT alignment of the initial query seqs to build the HMMR profiles), respectively, and re-aligned in MAFFT. After visual inspection for gap-causing sequences, such sequences were removed using the Phyfocus subprogram Alignment Editor (*w* = 347, *g* = 0.9). Finally, the remaining sequences were re-aligned in MAFFT and used in IQ-Tree (m = MFP + C60, alrt = 1000, bb = 1000 nt = 24) to construct the final phylogeny.

### Hox gene cluster annotation

The same coverage criteria applied to whole genomes in the Hox peptide phylogeny were also applied to annotated chromosomes and scaffolds used for Hox gene annotation. Full chromosomes from assemblies were obtained from *D. melanogaster* (NT_033777.3), *Bombyx mori* (NC_051363.1), and *Tribolium castaneum* (NC_007417.3). As non-holometabolan hexapod assemblies are frequently assembled only to the scaffold level, qualifying genomes were also examined to see if Hox loci could be identified on one scaffold, or on multiple scaffolds with near 100% identity overlaps that justify concatenation. The non-insect hexapod *Folsomia candida* contained all predicted Hox loci on one scaffold (NW_019091196.1), while the Hox loci for *Ephemera danica* were identified on two scaffolds with > 99.8% overlap identity when aligned using MAFFT v7.505, FFT-NS-1 method (KZ497623.1 and KZ497756.1; Additional file 2: Fig. S1). Hox loci for *C. dipterum* were on three scaffolds that could not be confidently concatenated and were thus excluded. All Hox gene annotations were based on protein annotations provided by NCBI, and thus represent the coding sequence for each gene. Hox gene clusters were plotted using SnapGene Viewer (v6.0.2; Insightful Science, snapgene.com) then visualized in Keynote (v9.2.1).

## Results

### *H. limbata* read & assembly statistics

Sequenced paired-end raw reads were 151 base pairs in length. Phred-based quality scores for read pairs differed dramatically, with the forward R1 reads having a mean Phred score of 33 (99.95% call accuracy) or greater across all bases, while the reverse R3 reads had mean Phred scores that varied extensively, from 22 (99.37%) or greater initially, but dropping rapidly after base pair 64 down to 2 (36.90%) by base pair 90 (Additional file 1: Figs. S3 and S4). No reads were flagged as poor quality. While the ORP and CLC assemblies of *H. limbata* produced a similar number of contigs, the ORP assembly produced generally longer contig sequences (Table 1). The two assemblies also differed notably in terms of quality, with the ORP producing far higher TransRate and BUSCO completion scores (Table 1). Given the superior quality of the ORP assembly, open reading frame (ORF) annotation and alignments were done exclusively with ORP contigs. The only two exceptions are *H. limbata* lab**,** which is derived from a CLC contig, and *H. limbata* Dfd, which used the consensus from combining an ORP and CLC contig (Additional file 2: Fig. S2). The ORP assembly was annotated against *Folsomia candida*, *Nilaparvata lugens*, *Zootermopsis nevadensis* and UniRef90 peptide databases (Table 1; [59]).

**Table 1:** Assembly and quality metrics for ORP and CLC transcriptomes of *H. limbata* reads

Criteria	ORP	CLC
Number of contigs	83,795	93,561
Number of contigs with ORFs	15,543	16,100
Number of annotated contigs	25,134	NA*
Mean contig length	702	529.36
Longest contig	26,091	22,280
Shortest contig	201	82
TransRate Assembly score	0.215	0.022
TransRate Optimal score	0.243	0.035
BUSCO Complete Score	1213 (88.8%)	839 (61.4%)

The number of contigs with ORFs was provided by TransRate. The TransRate assembly score assesses how accurate and complete an assembly is, while the optimal score is the assembly score obtained after removing all poorly assembled contigs. The complete BUSCO score represents the number of database sequences identified in the assembly, and was calculated using the insect ortholog database Odb10

^*^The number of annotated ORFs was determined via Diamond annotation and was not assessed for CLC contigs

### Hox peptide annotations

We identified proteins for eight of the ten canonical Hox genes in *H. limbata, C. dipterum,* and *E. danica* (see Additional file 1: Table S1 for accession values, Additional file 3 for fasta sequences). Additional Hox protein sequences for *H. limbata* Ubx and Abd-A and *C. dipterum* Pb, Dfd, Antp, and Abd-A, were identified (Additional file 2: Figs. S3–S8), along with putative Zen and Ftz proteins in *E. danica* (Additional file 2: Figs. S9–S10). A possible Ftz homeodomain was also identified within the putative Scr homolog for *C. dipterum* (Additional file 2: Fig. S11), making this a chimeric sequence. Excluding hypothetical proteins, all top reciprocal BLASTp hits were for the eight identified Hox proteins, with *e* values ranging from 3E-53 to 2E-104 for *H. limbata*, 2E-28 to 1E-96 for *E. danica*, and 4E-52 to 2E-103 for *C. dipterum* (Additional file 1: Tables S2, S3, and S4). Of the eight *H. limbata* Hox sequences, all but *lab, Dfd*, and *Abd-B* code for complete protein sequences.

**Fig. 1:**
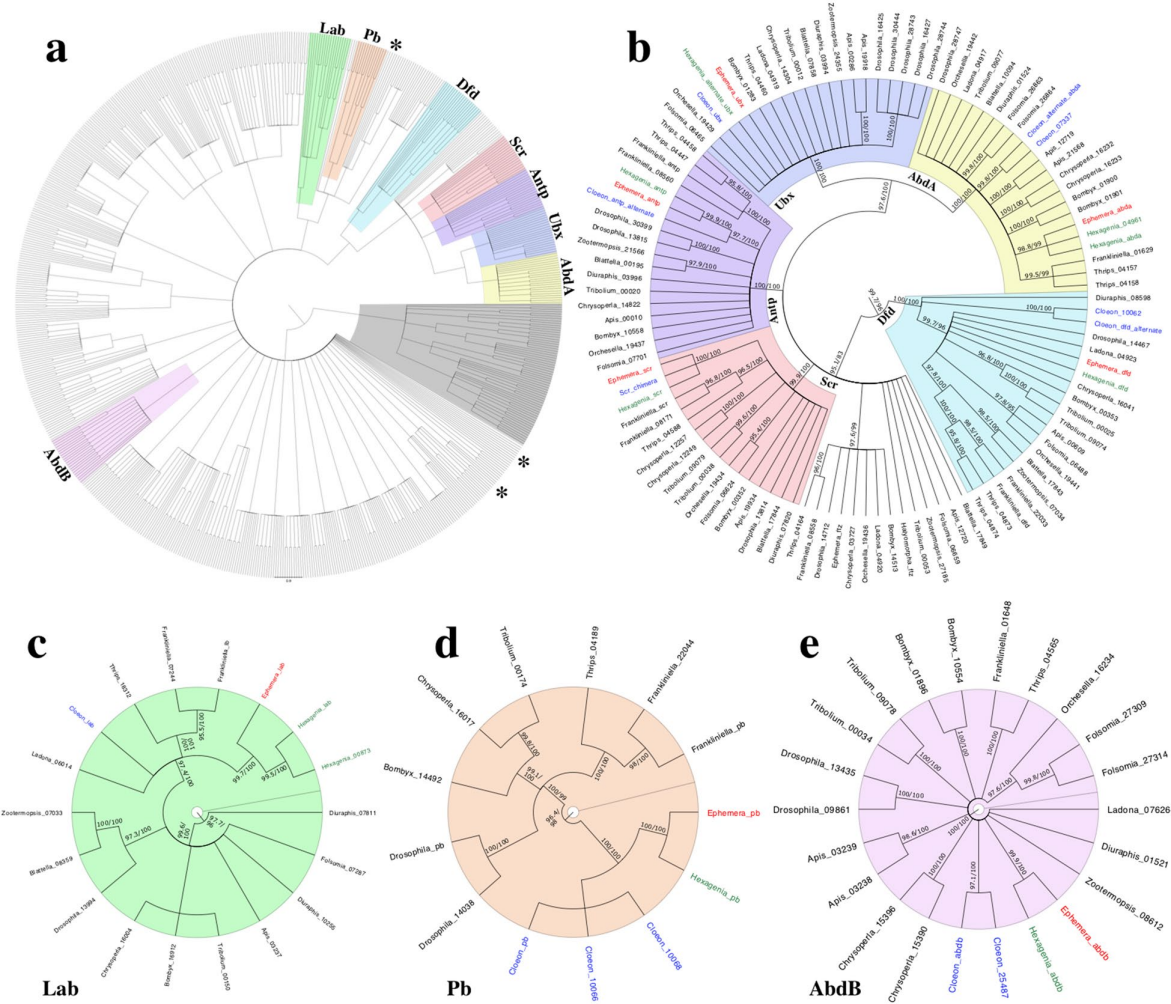
Phylogeny of hexapod homeodomain proteins. **a** Phylogeny of all ANTP class homeodomain proteins obtained from 16 hexapod species, and rooted with non-ANTP class homeodomain proteins (grey) as an outgroup. Clear monophyletic clades for Zen and Ftz were not identified, with query homologs and putative *E. danica* sequences for Zen sorting individually (asterisks denote approximate location). **b**–**e** Mayfly “genus_protein” tip labels are colored as follows: *H. limbata* (green), *E. danica* (red), *C. dipterum* (blue). **b** Subtree depicting the monophyletic groups for Dfd, Scr, Antp, Ubx, and Abd-A. Putative mayfly Hox sequences sort into each monophyletic clade. Note that the series of unsupported branches (not colored) bear some known Ftz homologs, including *E. danica* Ftz, and may represent an unsupported Ftz clade. **c**–**e** Subtrees for the Lab, Pb, and Abd-B monophyletic groups, respectively. In each, putative Hox sequences for all three mayfly species examined are present. For all trees, Monophyletic Hox protein clades are highlighted as follows: Lab (green), Pb (orange), Dfd (sky blue), Scr (red), Antp (purple), Ubx (blue), Abd-A (yellow), Abd-B (pink). All nodes with UltraFast Boostrap values less than 95 and SH-aLRT values less than 80 were collapsed into polytomies

Phylogenetic analysis of all homeodomain proteins identified in the *E. danica* and *C. dipterum* genomes and *H. limbata* transcriptome placed members of the eight identified mayfly Hox proteins in their respective monophyletic groups, further supporting their identification (Fig. 1a–e). Supported monophyletic groups for Zen and Ftz were not identified for any insect species. Possible Ftz homologs receiving insufficient support for a monophyletic group are present in the larger Hox monophyletic subclade (Fig. 1b), while the putative *E. danica* and two query Zen homologs sort independently of each other in the overall phylogeny (Fig. 1a).

Most identified *H. limbata* Hox peptides have functional domains and motifs that are highly conserved with those of other hexapods and provide the basis for describing mayfly Hox sequences here; while the putative Hox proteins for *E. danica* and *C. dipterum* are often less complete than their *H. limbata* homologs, they frequently shared these conserved regions as well. The *H. limbata* homeodomains (HD) are highly conserved, with identities of over 93% compared to homologs from other hexapods (Additional file 2: Figs. S12–19). The hexapeptide (Hx) motif was identified in seven of the eight *H. limbata* Hox proteins (Additional file 2: Figs. S12–18) and contained a core YKWM (Fig. S12) or YPWM (Additional file 2: Figs. S13–18) sequence. The *H. limbata* Abd-B protein lacks the Hx motif, and instead has only one conserved tryptophan residue, similar to that of other hexapod homologs (Additional file 2: Fig. S19). Including this conserved tryptophan, all eight *H. limbata* Hox proteins contained a linker region (LR) between the Hx and HD that was longest in the anterior Hox proteins (Additional file 2: Figs. S12–S13, 69 and 15 residues long, respectively), and progressively shorter in most posterior peptides (e.g., Additional file 2: Figs. S15 and S16, 14 and 4 residues long, respectively), with three semi-conserved residues in the Abd-B homolog (Additional file 2: Fig. S19).

Specific subgroups of Hox proteins contain additional conserved motifs. At the N-terminus is the SSYF motif found in Scr, Antp, Ubx, and Abd-A peptides (Additional file 2: Figs. S15–18). As the N-terminus arm is missing in our partial *H. limbata* Dfd peptide, it is unclear if H. *limbata* Dfd contains the SSYF motif as do other hexapod homologs (Additional file 2: Fig. S14). Ubx and Abd-A contain a number of unique signatures identified in *H. limbata*, including the UbdA peptide, the QAQA and Poly-A sequences unique to Ubx, and the TDWM and PFER motifs unique to the Abd-A linker region (Additional file 2: Figs. S17–18).

### Mayfly Hox Cluster Organization

**Fig. 2:**
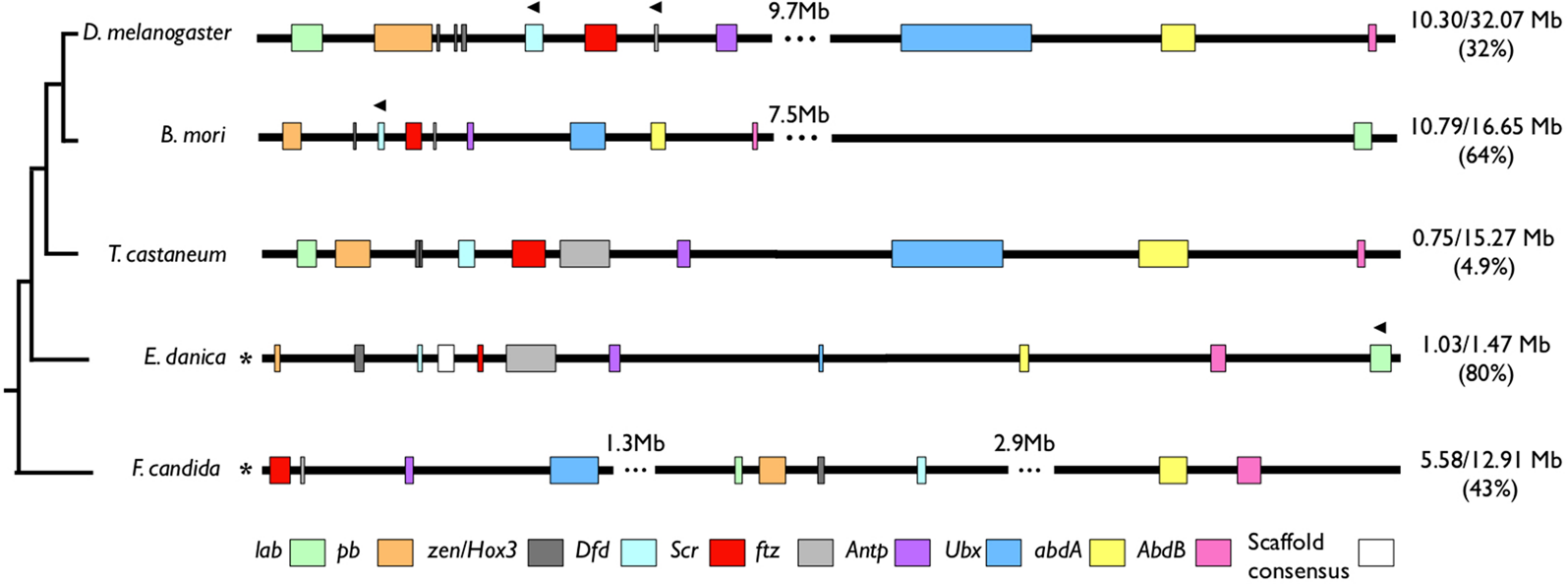
Genomic organization of the Hox gene cluster in a sampling of Hexapod species. Hexapod species phylogeny is after [25]. Scaffold-level sequences are annotated with a (*) after the species; all others are chromosome level. Ellipses within the sequences denote excluded genomic sequence, with the values above them denoting excluded sequence size. Hox genes are annotated as colored boxes: *lab* (green), *pb* (orange), *zen* (dark grey), *Dfd* (sky blue), *Scr* (red), *ftz* (light grey), *Antp* (purple), *Ubx* (blue), *abd-A* (yellow), *Abd-B* (pink). Note that the *E. danica* sequence is a concatenation of two scaffolds, the consensus overlap of which is denoted by a white box. Black arrowheads above Hox genes show sequences with opposite gene orientation. Values to the right of each sequence represent the size of the sequence shown, including ellipses, and its percentage in regard to the sequence’s full size. Accession values for the scaffolds and chromosomes used are provided in Additional File 1: Table S1

The genomic distance between different Hox coding sequences varies extensively between hexapod species, with some insects like D*. melanogaster* and *B. mori* having sizable distances of over 7 Mb between anterior and posterior Hox genes, or smaller but more frequent distances as seen in the non-insect hexapod *F. candida* (Fig. 2). The *E. danica* Hox cluster more closely resembled *T. castaneum* in having tightly grouped coding sequences that span approximately 1 Mb, with no notable distances between any particular Hox genes. Hox transcriptional orientation and overall order for *E. danica* appears similar to that of other insects, with the exception of *E. danica lab*, which has a different orientation and is located after *Abd-B*, similar to *B. mori lab* (Fig. 2).

### Expression of Antp, Ubx, and Abd-A proteins during *H. limbata* embryogenesis

**Fig. 3:**
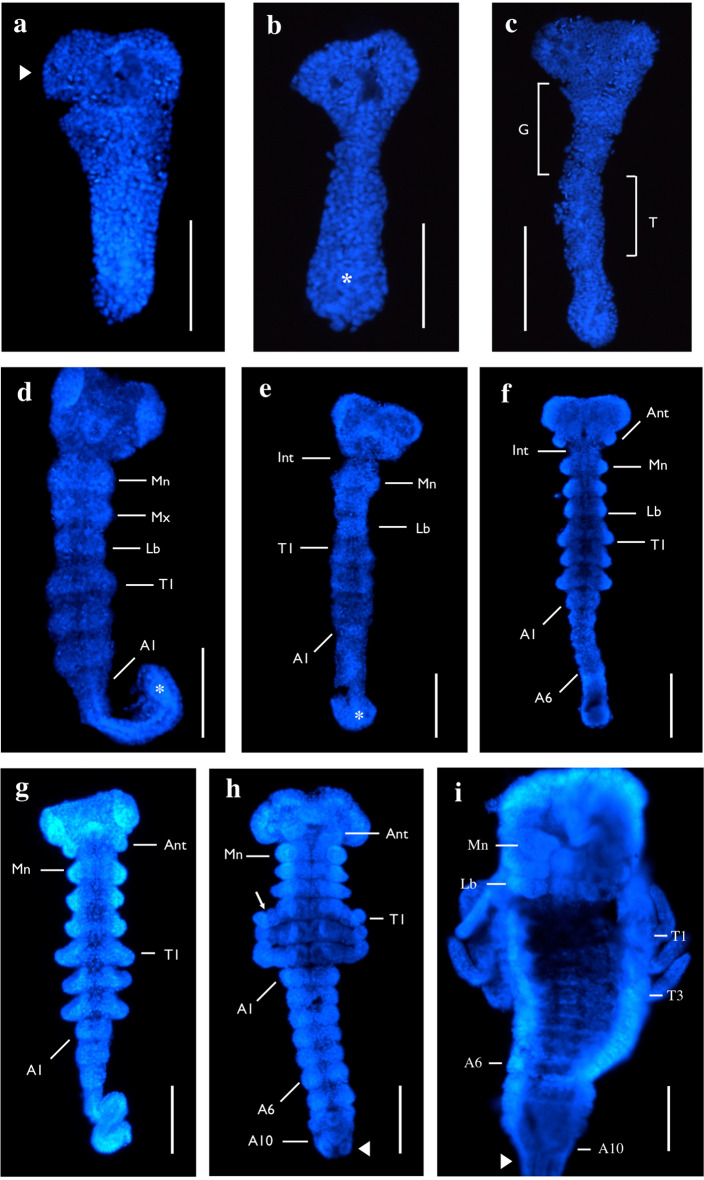
Nuclear DAPI staining of embryogenesis in *H. limbata* embryos. Ventral view, anterior to the top in all panels. **a**–**d** Early embryos. **a** *H. limbata* begin ontogeny with a defined ocular region (white arrowhead) but no clear segmentation. **b** Segmentation remains indistinct as the germ band continues extending via the posterior elongation zone (asterisk), which is readily identifiable as a wider bulge at the posterior end of the embryo. **c** Segments gradually become visible in the presumptive gnathal (G, white bracket) and thoracic (T, white bracket) regions. **d**, **e** Mid stage embryos. All pre-abdominal segment borders are clearly demarcated, while abdominal segments begin developing in a sequential fashion at the extending germ band (asterisk). **f**, **g** Late stage embryos; in g, the posterior abdomen is folded laterally. Late stage embryos develop most of the 10 abdominal segments, while gnathal and thoracic limb bud development (**f**) and elongation (**g**) becomes prominent. **h**, **i** Oldest staged embryos have clearly distinguishable body segments. In younger embryos of this stage (**h**), segmentation is visible in the thoracic limbs (white arrow); the abdominal segments also become wider, and preliminary terminal filaments (white arrowhead) are clearly present at the posterior end of the abdomen. **i** Oldest embryos are notably wider throughout the anterior–posterior axis, have clearly jointed gnathal and thoracic appendages, and bear developing terminal filaments (white arrowhead). Anterior is at the top in all panels. *Scale bars* 0.10 mm. Image magnifications are 100X for e–h, 200X for **a–d** and **i**

**Fig. 4:**
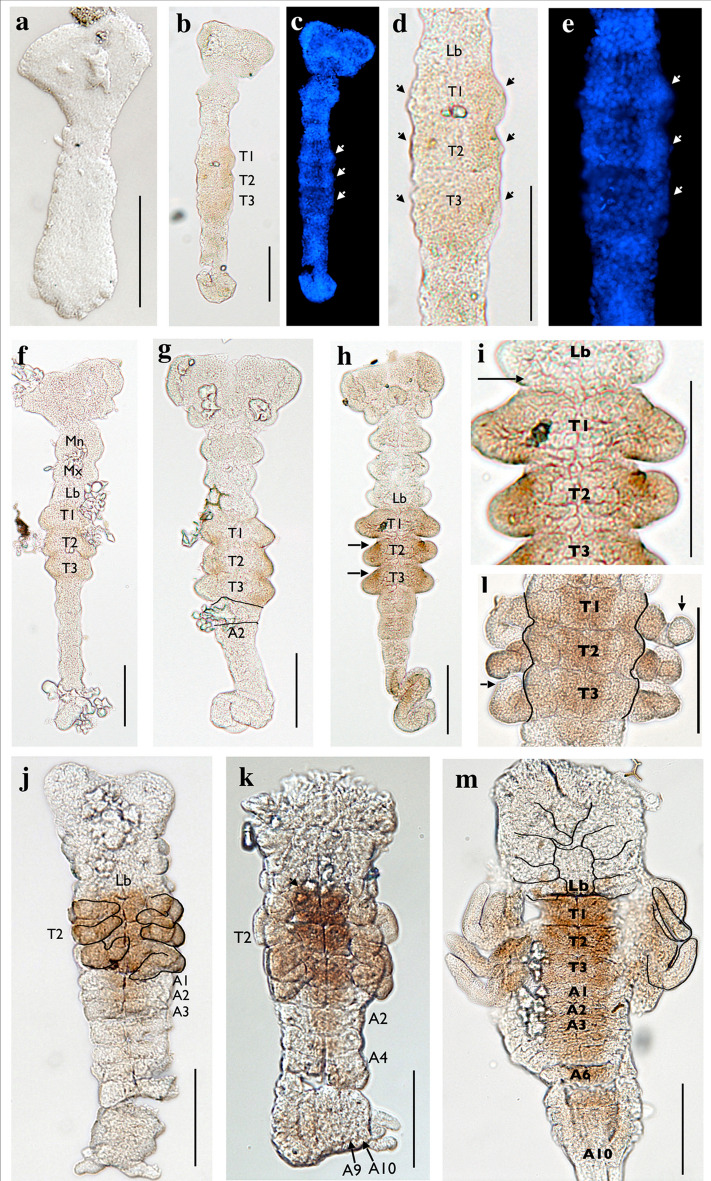
Horseradish peroxidase staining of *H. limbata* embryos using the Antp (4C3) antibody (**a**, **b**, **d**, **f**–**m**), with select corresponding DAPI stains (**c,** **e**). Early embryos do not show any expression (**a**). As the embryo adds segments to the posterior, expression is present in the three thoracic segments, where thoracic limb buds will eventually protrude (**b**, arrows in **c** and magnified views in **d**, **e**). As the limb buds elongate, expression extends into the buds (**f**, **g**), eventually appearing as patches of strong expression at the anterior of T2 and T3 (arrowheads in h, magnified view in i), while expression at the midline of each segment is less strong (**h**, **i**). At this time, expression also begins to faintly appear on the posterior edge of the labial segment (**h**, arrow in **i**). After segmentation concludes, strong expression in the thorax and thoracic limbs is present, weak expression is still evident at the posterior edge of the labial segment, and expression is seen in the center of the A1–A3 segments (**j**, **k**). Thoracic limb expression is strongest in the proximal region and grows fainter at the distal tips (arrows in l). In the oldest embryos we imaged, expression in the abdomen becomes stronger and extends up to the A10 segment, but is absent from the lateral edges (**m**). Expression at this stage extends from the posterior of the labial segment through the A10 segment (**m**). Mn, mandible; Mx, maxilla; Lb, labium; T1–T3, thoracic segments; A1–A10, abdominal segments. Ventral view, anterior to the top in all panels. *Scale bars* 0.10 mm. Image magnifications are 200X for **a**, **d**, **e**, and **j**–**m**, 100X for **b**, **c** and **f**–**i**

During embryogenesis, mayflies sequentially develop additional abdominal segments in a manner similar to that observed in short and intermediate germ insects (Fig. 3). Early *H. limbata* embryos do not show Antp expression (Fig. 4a). Once the precursors to the gnathal and thoracic segments form, weak expression appears in the three thoracic segments, especially along the sides where thoracic legs will develop (Fig. 4b, d). As the thoracic limb buds appear, expression becomes prominent at their edges (Fig. 4f–h), with midline expression present but weaker (Fig. 4f–h, magnified in 4i). Expression in post-segmentation embryos becomes stronger in the thoracic midline, and extends through the abdominal segments beginning in A1–A3 (Fig. 4j), then through to A9 (Fig. 4k). Thoracic limb expression remains present in the proximal portion of the limb, and is absent at the distal tips (Fig. 4l). In late-stage embryos (Fig. 4m), abdominal expression becomes uniform across the thorax and abdomen, but is still absent from the abdominal lateral edges and the A10 segment (Fig. 4m).

**Fig. 5:**
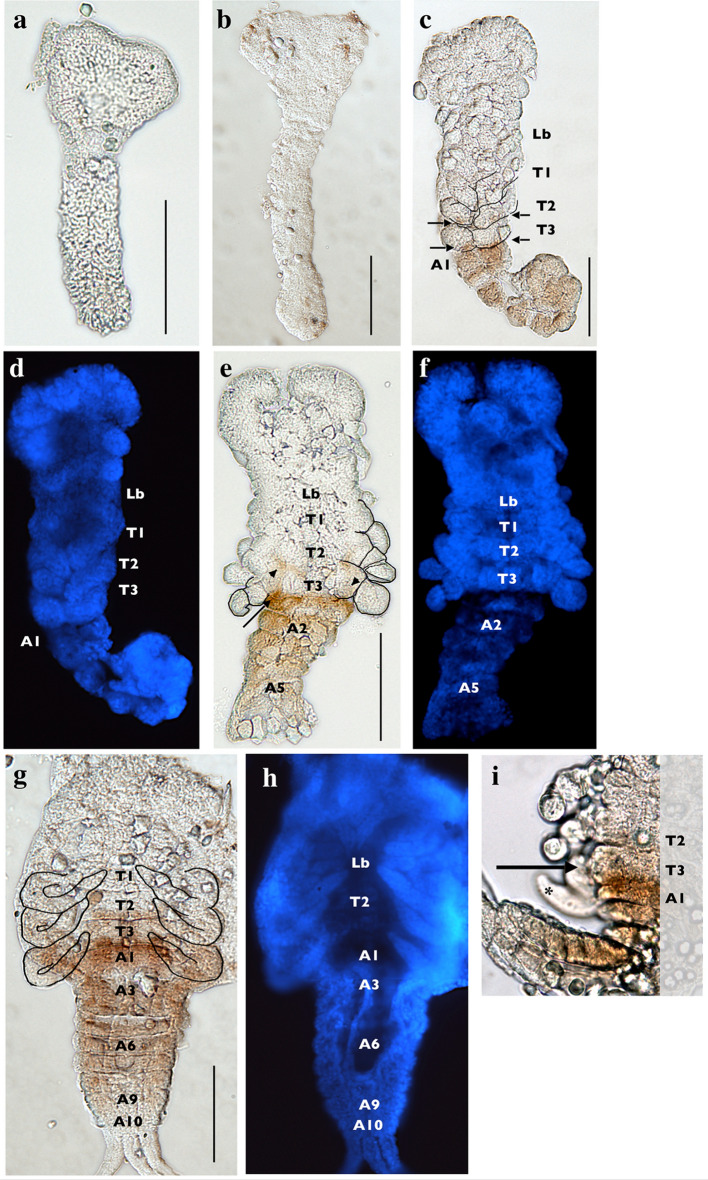
Horseradish peroxidase staining of *H. limbata* embryos using the Ubx/Abd-A (FP6.87) antibody (**a**–**c**, **e**, **g**, **i**), with corresponding DAPI stains (**d**, **f**, **h**). Early in embryogenesis, expression was not visible (**a**, **b**). The first apparent expression was observed in post-segmentation embryos as small posteriolateral patches in the T2 and T3 segments (arrows in **c**). Abdominal expression was stronger and extended through all abdominal segments until A10, with the strongest staining found in the A1 segment (**c**). Weak thoracic staining continued to appear in older embryos at the posteriolateral edges of T2 and T3 (arrowheads in **e**), with stronger expression in the lateral regions as compared to the midline. In addition, expression was strong at the posterior edge of T3 and the anterior edge of A1 (e, arrow). In the oldest embryos imaged, expression was present along the posterior boundaries of T2 and T3 (**g**), strongest at the posterior of T3 and anterior of A1, and remained through the abdomen, reducing in intensity toward the posterior segments (**g**). The posteriorolateral thoracic staining observed in segmented stages appears to extend close to the developing limbs, and possibly into the proximal limb structure (**i**, arrow). Staining is entirely absent from the distal tips of thoracic limbs (**i**, asterisk). T1–T3, thoracic segments; A1–A10, abdominal segments. Ventral view, anterior to the top for images **a**–**h**; lateral view, anterior to top in image **i**. *Scale bars* 0.10 mm. Magnifications are 200X for all images

Like Antp, Ubx and Abd-A expression is not evident in early *H. limbata* embryos (Fig. 5a), including those that have nearly developed the presumptive gnathal and thoracic segments (Fig. 5b). Once segmentation is complete, expression is prominent throughout all abdominal segments except for A10, with weak expression appearing at the posterior edges of T2 and T3 (Fig. 5c). Thoracic expression becomes more prominent in post-segmentation embryos (Fig. 5e), with expression beginning to spread in T3 and intensifying where the T3 and A1 segments meet. In the oldest embryos imaged (Fig. 5g), these expression patterns persist, with strong expression at the meeting edges of T3 and A1, and expression evident along the entire posterior edge of T2. In all documented stages, expression is not observed in the A10 segment. Unlike Antp expression, lateral expression in the segments is strong, while midline expression in the thorax (Fig. 5e) and abdomen (Fig. 5e, g) is reduced. It is unclear whether the lateral thoracic expression (Fig. 5c, e, g, i) is present only in the body segment, or if it extends to proximal compartments of the limb; however, staining is absent in the distal portions of the limb (Fig. 5i).

## Discussion

### ORP assembly quality is comparable to assemblies of non-model insects

Our 88.8% ORP BUSCO completion score was similar to or higher than those seen in recently published insect transcriptome data (e.g., [60–62]*.*). Likewise, our ORP TransRate assembly score of 0.215 is within the range of scores currently reported for insect transcriptome data (e.g.*,* [63, 64]), and is of higher quality than nearly 50% of transcriptomes deposited in the NCBI TSA database as of 2016 [47]. The TransRate assembly score is significantly impacted by both read quality and read duplication during PCR amplification [48]. Thus, the low quality seen in many of our R3 reads may have depressed the assembly score despite read trimming done by the ORP. These factors also highlight the importance of read trimming in assembly quality, as trimming was only conducted for the ORP assembly.

### Mayfly Hox sequences are highly conserved relative to other hexapods

The loci order of the mayfly Hox cluster is largely similar to that classically identified in both *Drosophila* and many insects [8–10]; however, *E. danica lab* is located after *Abd-B*. Though extensive distances between Hox genes are absent, this organization resembles that identified in *Bombyx mori* [65]. The Hox proteins themselves also show extensive similarities with other insects, with the eight identified Hox proteins from all three mayfly species phylogenetically sorting amongst their putative homologs, and most containing the homeodomain, linker region, and hexapeptide motif widely conserved in most insect Hox proteins. Several functional regions specific to particular Hox proteins are also present in the *H. limbata* homologs. These include the presence of an N-terminal SSYF motif in Scr, Antp, Ubx, and Abd-A, and its absence in Lab and Abd-B; TDWM and PFER motifs in the linker region of Abd-A; the C-terminal UbdA peptide in both Ubx and Abd-A; and C-terminal QAQA and poly-A sequences in Ubx [20].

Further evidence of high sequence conservation in *H. limbata* Hox proteins comes from specific residues within these functional regions. For example, there are four residues unique to Hox homeodomains: a glutamic acid in alpha-helix 1, an arginine and glutamic acid in alpha-helix 2, and a methionine in alpha-helix 3 [20]. These residues were all identified in our *H. limbata* sequences (e.g*.*, residues Glu-136, Arg-148, Glu-150, and Met-171 in Additional file 2: Fig. S12; see also Additional file 2: Figs. S13–19 and [20]). Additional protein-specific homeodomain residues exist; homeodomains for lab and pb have the largest number of unique residues, primarily within the N-terminal arm and first and third alpha-helices [20].

Three residues unique to the homeodomain N-terminals of Antp, Ubx, and Abd-A (e.g., Gly-22, Gln-24, and Thr-25 in Additional file 2: Fig. S16; see also Additional file 2: Figs. S17–18) and Abd-B (Lys-12, Lys-13, and Pro-16, Additional file 2: Fig. S19) [20], were identified in the corresponding *H. limbata* homologs. The SSYF and hexapeptide motifs are likewise present in the putative *H. limbata* Hox peptides.

A number of residues in the linker regions of Lab, Pb, Dfd, and Scr proteins are also conserved, though these vary more than the homeodomain and hexapeptide regions. For example, many Lab linker regions in metazoans share a VKRXXPKTXKXE sequence [20], which in *H. limbata* is represented by VKRXXPKP (Additional file 2: Fig. S12, residues 7–14, with the conserved threonine replaced by proline). The rest of the linker sequence varies in most aligned hexapods, a phenomenon that is also seen in the conserved XKKXXK sequence for Pb (Additional file 2: Fig. S13, residues 7–13), and the KVHL sequence in Dfd and Scr (Additional file 2: Figs. S14–S15, 4, residues 11–14 and 11–15, respectively; [20]).

While the eight identified Hox proteins appear extensively conserved, protein coding sequences for *zen* and *ftz* were not identified in the early nymphal transcriptome of *H. limbata* and could not be reliably identified in monophyletic clades within our phylogeny, despite using mayfly genomic data. While the inability to identify these proteins in *H. limbata* could be due to appreciably low levels of transcription of these genes during early nymphal stages, their limited identification in the two mayfly genomes suggests some inherent difficulty with homolog assignment, perhaps due to their evolutionary diversity. Such difficulty is reasonable as *zen* and *ftz* are regarded as “rogue” Hox genes that underwent extensive diversification within ecdysozoan evolution [11, 66] and no longer function as traditional Hox genes in many insects. Given the variable evolution of these genes, the presence of possible *zen* and *ftz* homologs in *E. danica*, and the possible Ftz homeodomain within *C. dipterum* Scr, it is evident that homologs for these Hox genes are present in mayflies but difficult to confidently identify, perhaps due to both variability from known homologs and issues with distinguishing Zen and Ftz sequences from other homeodomain proteins.

### Antp, Ubx, and Abd-A embryonic expression is highly conserved amongst insects

Expression of Antp, Ubx, and abd-A during *H. limbata* embryogenesis closely resembles that of other insects, particularly non-holometabolous species. In the case of *H. limbata* Antp, we documented expression primarily through the embryonic thorax and abdominal midline. During segmentation in *D. melanogaster*, Antp expression occurs from the posterior of the labial segment to the abdominal segments, with the strongest expression in the thorax. During germ band retraction, the concentration of *Antp* transcripts and proteins remains strongest in the thorax, while abdominal expression is limited to the midline [67–69]. Most studies of *Antp* gene products in holometabolous (*Apis mellifera*, [70]) and non-holometabolous species (*Schistocerca americana,* [68]; *Gryllus bimaculatus*, [71]) reveal an anterior expression boundary in posterior cells of the labial segment, as we observed in *H. limbata*. Similarly, Antp expression in *H. limbata* occurs throughout the thorax and midline of the abdominal segments and closely matches that of other holometabolous and non-holometabolous insects, though some species show lateral staining of transcripts in the abdominal tracheal pits [70]. The reduced midline thoracic staining and stronger proximal staining of *H. limbata* thoracic limb buds is also observed in orthopteran *Antp* gene products [68, 71], providing further evidence that Antp expression is highly conserved between *H. limbata* and other insects, particularly non-holometabolan species.

Similar to Antp expression, Ubx and Abd-A expression is highly conserved between *H. limbata* and other insects despite the distinct differences in development between many holometabolous and non-holometabolous species. Combined Ubx and Abd-A expression was strongest, where the T3 and A1 segments meet, and along the lateral portions of the A1–A8 abdominal segments, with weaker expression from A8–A10. In *D. melanogaster*, Ubx and Abd-A show largely overlapping and complementary expression profiles. *D. melanogaster* *Ubx* gene products are expressed before segmentation in the presumptive T3 and A1–A7 segments, particularly at the T3 and A1 juncture and within the anterior portion of each segment; this pattern persists after complete segmentation, with additional expression along the abdominal midline and weakly in A8 [72–74]. After the development of all body segments, *D. melanogaster* Abd-A expression is seen nearly simultaneously within A1–A7, most strongly within the posterior of each segment; like Ubx, it later extends to the abdominal midline and into A8 [74–76]. Ubx and Abd-A expression is similar in the honeybee *Apis mellifera* but begins in A1–A4 before spreading through A1–A7 and does not extend to the abdominal midline [70], a highly conserved pattern seen in both *H. limbata* and many other insects. In the orthopteran *Gryllus bimaculatus*, *Ubx* transcripts are first expressed in the posterior growth zone and in the presumptive T3, with expression after segmentation strongest at the T3 and A1 juncture [71]. *Ubx* expression in the apterygote *Thermobia domestica* is similar, but also extends anteriorly around the T2 and T3 limb buds during germ band extension, similar to *H. limbata* lateral staining in the T2 and T3 segments [78]. The extension of Ubx and abd-A lateral expression through the developing abdomen until A10, followed by a post-segmentation weakening of expression from A8–A10, is widely conserved between *H. limbata* protein expression and the gene products of other insect taxa [71, 78].

### Models of Antp, Ubx, and Abd-A function in mayflies

The body plan of mayfly nymphs diverges from that of most other insects in possessing unique limb like abdominal appendages. Regulation of thoracic limb and abdominal appendage development in insects is controlled by the Hox proteins Antp, Ubx, and Abd-A, each of which contain functional regions well conserved between *H. limbata* and many insects. These include the SSYF motif necessary for the transcriptional activation of downstream target genes [77] and the hexapeptide motif, which contributes to Extradenticle protein binding [79]. The length of each Hox gene linker region is likewise widely conserved between *H. limbata* and other insects, and facilitates proper gene function [20, 80]. However, as little is known regarding the functional significance of most linker region residues [20], the potential impact of both *H. limbata* specific and gene-specific differences in linker region sequences remains unknown. Conserved N-terminal residues of Antp, Ubx, and Abd-A were also identified in *H. limbata* and play a major role in specifying DNA binding affinity [20, 81]. Several functional regions are specific to Ubx and Abd-A, including the UbdA peptide, QAQA, and poly-A sequences; all three have been demonstrated in *Drosophila* to repress the gene *Distal-less (Dll)* [82–84], and are present in the identified *H. limbata* homologs. *H. limbata* Abd-A also contains the TDWM and PFER linker region motifs, which regulate Extradenticle binding and *wingless* transcription, respectively [85, 86].

At the phenotypic level, Antp promotes leg development in the thorax [87] and is abdominally expressed as part of the developing central nervous system in both insects [67, 68, 70, 71, 88] and crustaceans [89]. In a number of holometabolan insects like *D. melanogaster*, Ubx and abd-A prevent abdominal limbs from developing through inhibition of *Dll* [90], which specifies the distal portion of developing appendages [91, 92]. In other insects such as coleopterans and orthopterans, Ubx serves as an appendage modifier that is co-expressed with Dll in the A1 segment, resulting in pleuropod development during embryogenesis [93, 94] and leaving abdominal limb repression primarily to Abd-A. Taken with our embryonic expression data, the shared functional regions between studied insects and *H. limbata* suggest similar roles for Antp, Ubx, and Abd-A during mayfly embryogenesis, leading to the development of thoracic limbs and an appendage-less abdomen during the first nymphal instar.

Our identification of putative Hox homologs in the transcriptome of early nymphal instars demonstrated that most Hox genes, including *Antp*, *Ubx*, and *abd-A*, continue to be expressed post-embryonically. As gills in *H. limbata* develop in the second instar ([95, 96], Additional file 1: Fig. S1), expression of these Hox genes may impact gill development. However, it remains to be seen where nymphal Hox expression occurs, and if predicted embryonic functions such as abdominal limb repression continues in nymphal stages. Insects that develop some form of abdominal appendages or appendage-like structures provide hypothetical models of how nymphal Hox expression can impact mayfly gill development. In one model, Ubx and/or Abd-A continue to have limb-repressive roles, requiring their expression patterns to be modified for gill development. This is seen in lepidopterans, which require both the repression of Ubx and Abd-A, and the expression of Antp and Dll in abdominal limb primordia, for the larval prolegs to develop [97–99]. However, it is also possible that the functions of mayfly Ubx and/or Abd-A are not entirely repressive. In coleopterans and orthopterans, Ubx serves as an appendage modifier that is co-expressed with Dll in the A1 segment, resulting in pleuropod development during embryogenesis [71, 93, 94]. Ubx and Abd-A may also not regulate gill development at all, particularly if gills are homologous to proximal appendicular structures, as some have hypothesized [3, 100]. Such a morphological distinction is seen in the firebrat *Thermobia domestica*, where Ubx and Abd-A expression do not appear to play a role in the development of styli on the A7–A9 segments, possibly because styli may be proximal structures not homologous to true appendages [78, 101]. In another example from sawfly embryos, abdominal prolegs develop despite both the abdominal expression of Ubx and Abd-A and the lack of abdominal Dll expression, suggesting that sawfly prolegs consist exclusively of morphologically proximal structures [99, 102]. Delineating which model is most applicable to mayflies requires both direct functional evidence for Antp, Ubx, and Abd-A in mayfly development and positional data on their expression during the first and second nymphal stages.

### Mayflies as a model system for EvoDevo research

Ephemeropterans and their life history have long been a focal point in ecologically focused research, both for their importance to freshwater ecosystems as a prey species and as a bioindicator of environmental health. Aside from phylogenetic assessments, mayflies have been far less prominent in the realm of evolutionary studies, particularly those from a developmental genetics perspective. Some of the first published expression data on mayfly developmental genetics regarded the spatial expression of *wingless*, Engrailed, *apterous*, and *vestigial* in the embryos of *Ephoron* mayflies [34, 35]. These works established IHC and ISH protocols within embryos and early nymphs of Polymitarcyidae mayflies. The development of the Baetidae *C. dipterum* as a model with transcriptomic and genomic sequence data, an established means of laboratory rearing, and functional IHC and ISH protocols [32] greatly expanded the tractability of mayfly models in evo-devo research.

Our annotated transcriptome and IHC protocol for *H. limbata* contributes similar resources for a North American Ephemeridae species. However, there are currently some limitations to spatial expression studies, such as the inability of several *Drosophila* based antibodies, including those specific to solely Ubx, Abd-A, or Abd-B, to produce valid staining (data not shown). Furthermore, attempts to adapt *Ephoron* ISH protocols to *H. limbata* using *abd-A* riboprobes produced staining similar to that of negative controls, preventing the effective use of transcriptomic sequence data [103]. The development of successful ISH protocols in *H. limbata* would open spatial expression options to both additional Hox genes and a wide array of possible segmentation and appendage patterning sequences putatively identified in the *H. limbata* data set [103].

Regardless of species, perhaps the most notable obstacle to developmental genetic research in mayflies is the lack of functional techniques, such as RNAi [32]. Attempts to develop RNAi in *H. limbata* faced mechanical issues with double-stranded RNA delivery, as the small size and durable chorion of *H. limbata* eggs created difficult constraints in terms of necessary needle diameter and strength [103]. Given the extensive resources that currently exist for *C. dipterum*, it is arguably the model of choice for developing gene function protocols in mayflies. Burrowing mayflies from Ephemeridae and Polymitarcyidae can broaden the phylogenetic, geographic, and lifecycle diversity of mayfly models, and provide additional sequence support for gene orthology identifications and comparisons in any mayfly species.

## Conclusions

Our investigation of mayfly Hox genes described the organization of the Hox gene cluster, consistently identified peptide sequences for eight of the ten canonical Hox genes, and revealed the presence of key functional regions highly conserved with other insect Hox homologs. The expression of embryonic Antp also is highly conserved with that of other insects, becoming apparent in the developing thoracic limb buds and subsequently spreading throughout the thoracic and abdominal midline. Similarly, embryonic Ubx and Abd-A expression closely matches what is observed in other studied insects, particularly non-holometabolan species. The extensive conservation of both *H. limbata* Hox sequences and expression profiles with those of other insects suggests that these Hox genes play a conserved role in specifying the thoracic limbs and appendage-less abdomen of first instar nymphs. The continued expression of these genes in first instars of *H. limbata* raises the possibility of Hox-regulated gill development; however, further progress in adapting spatial gene expression and function protocols for mayfly species is necessary to fully assess possible models of gill regulation and evolution. The increasing attention to mayfly developmental genetics and model status for evo-devo research has provided a number of crucial genomic and transcriptomic resources. With the present availability of annotated transcriptomic data, detailed Hox annotations, and molecular protocols tested across multiple species, there remain many exciting avenues for exploring the unique evolution and development of mayflies.

## Supplementary Information

**Additional file 1.** Includes all sequence accession values, background figures on mayfly development and image processing, and data on mayfly Hox reciprocal BLAST hits and *H. limbata* transcriptomic read quality.

**Additional file 2.** Contains all alignments generated in this study, including assessments of the mayfly Hox scaffolds and all identified mayfly Hox proteins.

**Additional file 3.** Contains all identified mayfly Hox protein sequences in FASTA format.

## Data Availability

*H. limbata* annotated Hox sequences have been deposited in NCBI's GenBank database under the accession numbers MZ773605–MZ773612, while the source sequencing reads are available in NCBI's SRA database via the accession number SRX6489924. ORP and CLC contig assemblies, ORP Diamond annotation results, and ORP Transdecoder peptides are available in the following Zenodo repository: https://doi.org/10.5281/zenodo.6624929. All other data generated or analyzed during this study are included or referenced in this published article and its supplementary information file.
