# Supplementary material for: Characterizing Hox genes in mayflies (Ephemeroptera), with Hexagenia limbata as a new mayfly model
Source: EvoDevo. 2022 Jul 27;13:15. doi: 10.1186/s13227-022-00200-w (PMC9331126; doi:10.1186/s13227-022-00200-w)
Supplement: Supplementary file 1 — Additional file 1. Includes all sequence accession values, background figures on mayfly development and image processing, and data on mayfly Hox reciprocal BLAST hits and H. limbata transcriptomic read quality. [file 13227_2022_200_MOESM1_ESM.pdf]

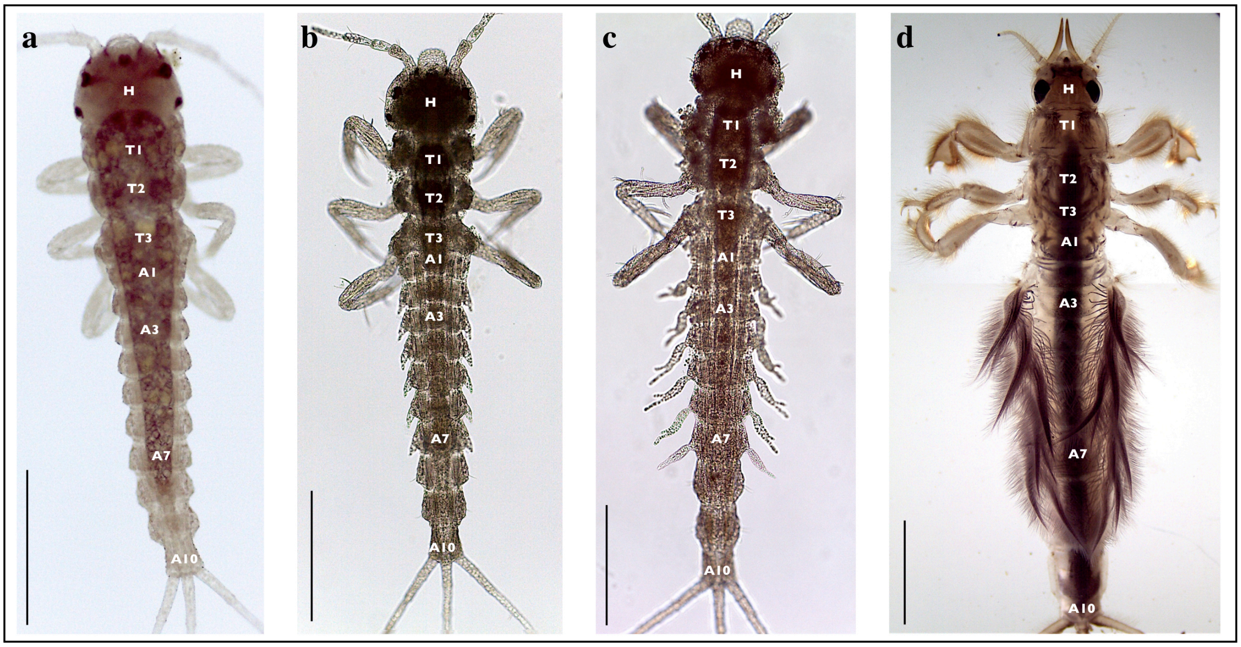

Additional file 1: Fig. S1 Morphological development of body segments in the first three (A-C) and an undetermined later (d) instars of *H. limbata*, dorsal views. (a) First instar hatchling, which lacks abdominal gills. (b-c) Abdominal gills first appear in the second instar on the A2-A7 segments, and elongate in the third instar. (d) After an undetermined number of instars, complete bilamellar abdominal gills are present on the A1-A7 segments. Scale bar is 0.25mm for (a-c), 3.0mm in (d).

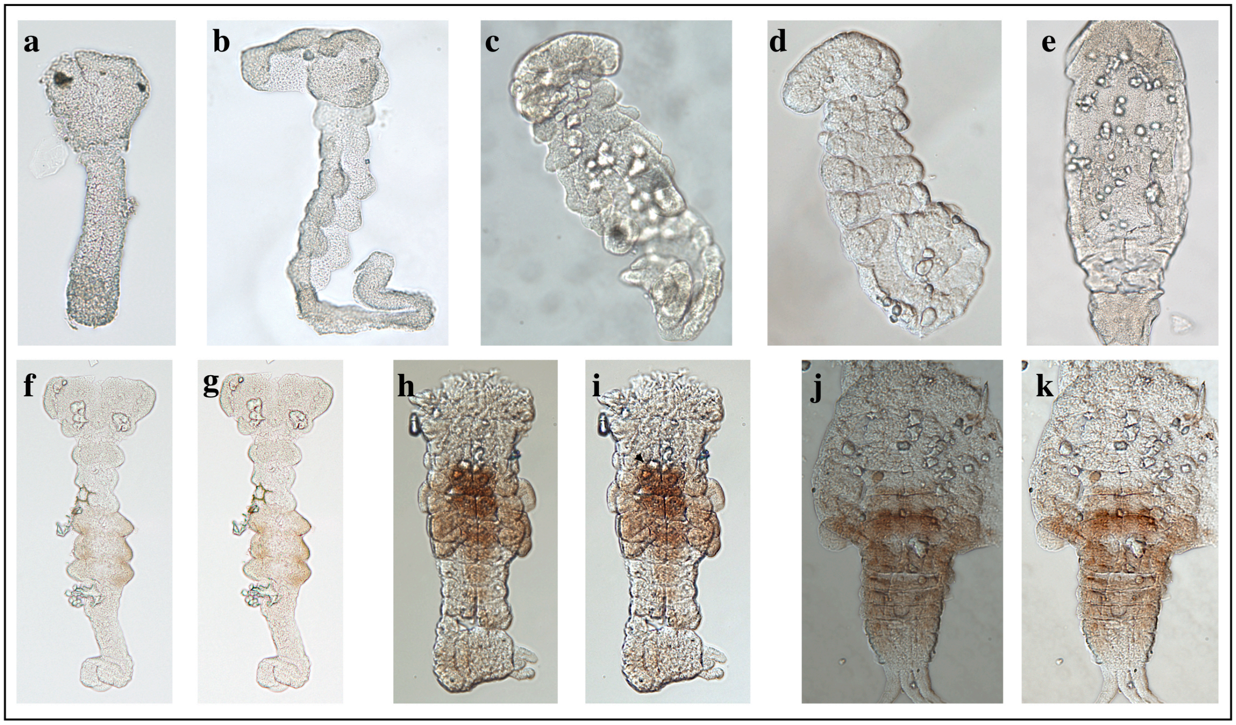

Additional file 1: Fig. S2 Negative controls (a-e) and image edit comparisons (f-k) for IHC data. IHC negative controls for Antp and Ubx/Abd-A studies lacked staining in early (a), mid (b-c), and late (d-e) stage embryos. Image editing entailed whole-photo adjustments to exposure, contrast, light balance, and color, as shown in the original (f, h, j) vs final (g, i, k) figure images.

Additional file 1: Table S1 Accession numbers and contig IDs for sequences in this study, organized according to use, data type, and species.

Use	Data Type	Species	Accession	Source
Assembly input	Reads	*Hexagenia limbata*	SRX6489924	NCBI
Multiple	CLC Contig Database	*Hexagenia limbata*	N/A	[1]
Multiple	ORP Contig Database	*Hexagenia limbata*	N/A	[1]
Multiple	Genome	*Ephemera danica*	GCA_000507165.2	NCBI
Multiple	Genome	*Cloeon dipterum*	GCA_902829235.1	NCBI
Diamond annotation	Genome	*Folsomia candida*	GCF_002217175.1	NCBI
Diamond annotation	Genome	*Nilaparvata lugens*	GCF_000757685.1	NCBI
Diamond annotation	Genome	*Zootermopsis nevadensis*	GCF_000696155.1	NCBI
Query Blast	Lab Peptide	*Drosophila melanogaster*	AAD19811	NCBI
Query Blast	Pb Peptide	*Drosophila melanogaster*	CAA45271	NCBI
Query Blast	Zen Peptide	*Drosophila melanogaster*	NP_476793.1	NCBI
Query Blast	Dfd Peptide	*Drosophila melanogaster*	AAD19796	NCBI
Query Blast	Scr Peptide	*Drosophila melanogaster*	AAD19795	NCBI
Query Blast	Ftz Peptide	*Drosophila melanogaster*	AAD19794	NCBI
Query Blast	Antp Peptide	*Drosophila melanogaster*	NP_996170	NCBI
Query Blast	Ubx Peptide	*Drosophila melanogaster*	AAF55355	NCBI

Use	Data Type	Species	Accession	Source
Query Blast	Abd-A Peptide	*Drosophila melanogaster*	AAF55359	NCBI
Query Blast	Abd-B Peptide	*Drosophila melanogaster*	NP_650577	NCBI
Query Blast	Zen Peptide	*Folsomia candida*	ABN42911.1	NCBI
Query Blast	Ftz Peptide	*Folsomia candida*	OXA55517.1	NCBI
Query Blast	Zen Peptide	*Nilaparvata lugens*	AUX14872.1	NCBI
Query Blast	Ftz Peptide	*Frankliniella occidentalis*	KAE8742594.1	NCBI
Query Blast	Zen Peptide	*Schistocerca gregaria*	CAB61208.1	NCBI
Query Blast	Ftz Peptide	*Tribolium castaneum*	NP_001034539.1	NCBI
Alignment	Lab Peptide	*Hexagenia limbata*	UJD73453.1	NCBI; CLC Peptide
Alignment	Lab Peptide	*Drosophila melanogaster*	AAD19811	NCBI
Alignment	Lab Peptide	*Tribolium castaneum*	EEZ99257.1	NCBI
Alignment	Lab Peptide	*Frankliniella occidentalis*	KAE8743597	NCBI
Alignment	Lab Peptide	*Ephemera danica*	KAF4518711.1	NCBI
Alignment	Lab Peptide	*Cloeon dipterum*	CAB3369110.1	NCBI
Alignment	Pb Peptide	*Hexagenia limbata*	UJD73454.1	NCBI; ORP Peptide
Alignment	Pb Peptide	*Drosophila melanogaster*	CAA45271	NCBI
Alignment	Pb Peptide	*Tribolium castaneum*	EEZ99256.1	NCBI
Alignment	Pb Peptide	*Frankliniella occidentalis*	KAE8751405.1	NCBI
Alignment	Pb Peptide	*Orchesella cincta*	ODM96207.1	NCBI
Alignment	Pb Peptide	*Ephemera danica*	KAF4522773.1	NCBI
Alignment	Pb Peptide	*Cloeon dipterum*	CAB3369103.1	NCBI
Alignment	Pb 10066	*Cloeon dipterum*	CAB3369102.1	NCBI
Alignment	Pb 10068	*Cloeon dipterum*	CAB3369104.1	NCBI
Alignment	Zen Peptide	*Drosophila melanogaster*	NP_476793.1	NCBI
Alignment	Zen Peptide	*Tribolium castaneum*	AAK16424.1	NCBI
Alignment	Zen Peptide	*Frankliniella occidentalis*	KAE8751404.1	NCBI
Alignment	Zen Peptide	*Folsomia candida*	ABN42911.1	NCBI
Alignment	Zen Peptide	*Ephemera danica*	KAF4522771.1	NCBI
Alignment	Dfd Peptide	*Hexagenia limbata*	UJD73455.1	NCBI; ORP & CLC Peptide
Alignment	Dfd Peptide	*Drosophila melanogaster*	AAD19796	NCBI
Alignment	Dfd Peptide	*Tribolium castaneum*	NP_001034510.1	NCBI
Alignment	Dfd Peptide	*Frankliniella occidentalis*	KAE8751403.1	NCBI
Alignment	Dfd Peptide	*Folsomia candida*	OXA55346.1	NCBI
Alignment	Dfd Peptide	*Ephemera danica*	KAF4522770.1	NCBI
Alignment	Dfd Peptide	*Cloeon dipterum*	CAB3369100.1	NCBI
Alignment	alt Dfd	*Cloeon dipterum*	CAB3369099.1	NCBI
Alignment	Dfd-grouping 10062	*Cloeon dipterum*	CAB3369098.1	NCBI
Alignment	Scr Peptide	*Hexagenia limbata*	UJD73456.1	NCBI; ORP Peptide
Alignment	Scr Peptide	*Drosophila melanogaster*	AAD19795	NCBI
Alignment	Scr Peptide	*Tribolium castaneum*	EEZ99252.1	NCBI
Alignment	Scr Peptide	*Frankliniella occidentalis*	KAE8742593.1	NCBI
Alignment	Scr Peptide	*Folsomia candida*	OXA56455.1	NCBI
Alignment	Scr Peptide	*Ephemera danica*	KAF4518724.1	NCBI
Alignment	Scr Chimera Peptide	*Cloeon dipterum*	CAB3372600.1	NCBI
Alignment	Ftz Peptide	*Drosophila melanogaster*	NP_477498.1	NCBI
Alignment	Ftz Peptide	*Tribolium castaneum*	NP_001034539.1	NCBI
Alignment	Ftz Peptide	*Halyomorpha halys*	KAE8573718.1	NCBI
Alignment	Ftz Peptide	*Folsomia candida*	OXA55517.1	NCBI
Alignment	Ftz Peptide	*Ephemera danica*	KAF4518722.1	NCBI
Alignment	Antp Peptide	*Hexagenia limbata*	UJD73457.1	NCBI; ORP Peptide
Alignment	Antp Peptide	*Drosophila melanogaster*	996170	NCBI
Alignment	Antp Peptide	*Tribolium castaneum*	NP_001034505.1	NCBI
Alignment	Antp Peptide	*Frankliniella occidentalis*	KAE8742595.1	NCBI
Alignment	Antp Peptide	*Orchesella cincta*	ODN05861.1	NCBI
Alignment	Antp Peptide	*Ephemera danica*	KAF4518721.1	NCBI
Alignment	Antp Peptide	*Cloeon dipterum*	CAB3372602.1	NCBI
Alignment	alt Antp Peptide	*Cloeon dipterum*	CAB3372603.1	NCBI

Use	Data Type	Species	Accession	Source
Alignment	Ubx Peptide	*Hexagenia limbata*	UJD73458.1	NCBI; ORP Peptide
Alignment	alt Ubx peptide	*Hexagenia limbata*	Gene.14755	ORP peptide; [1]
Alignment	Ubx Peptide	*Drosophila melanogaster*	AAF55355	NCBI
Alignment	Ubx Peptide	*Tribolium castaneum*	NP_001034497.1	NCBI
Alignment	Ubx Peptide	*Thrips palmi*	XP_034234416.1	NCBI
Alignment	Ubx Peptide	*Orchesella cincta*	CDI44537.1	NCBI
Alignment	Ubx Peptide	*Ephemera danica*	KAF4518720.1	NCBI
Alignment	Ubx Peptide	*Cloeon dipterum*	CAB3372607.1	NCBI
Alignment	Abd-A Peptide	*Hexagenia limbata*	UJD73459.1	NCBI; ORP Peptide
Alignment	Abd-A 04961	*Hexagenia limbata*	Gene.4959	ORP Peptide; [1]
Alignment	Abd-A Peptide	*Drosophila melanogaster*	AAF55359	NCBI
Alignment	Abd-A Peptide	*Tribolium castaneum*	EEZ99248.1	NCBI
Alignment	Abd-A Peptide	*Frankliniella occidentalis*	KAE8744407.1	NCBI
Alignment	Abd-A Peptide	*Orchesella cincta*	CDI44539.1	NCBI
Alignment	Abd-A Peptide	*Ephemera danica*	KAF4518716.1	NCBI
Alignment	Abd-A Peptide	*Cloeon dipterum*	CAB3366371.1	NCBI
Alignment	alt Abd-A Peptide	*Cloeon dipterum*	CAB3366369.1	NCBI
Alignment	Abd-A 07337	*Cloeon dipterum*	CAB3366370.1	NCBI
Alignment	Abd-B Peptide	*Hexagenia limbata*	UJD73460.1	NCBI; ORP Peptide
Alignment	Abd-B Peptide	*Drosophila melanogaster*	NP_524896	NCBI
Alignment	Abd-B Peptide	*Tribolium castaneum*	KYB29151.1	NCBI
Alignment	Abd-B Peptide	*Thrips palmi*	XP_034234521.1	NCBI
Alignment	Abd-B Peptide	*Orchesella cincta*	ODN02667.1	NCBI
Alignment	Abd-B Peptide	*Ephemera danica*	KAF4518714.1	NCBI
Alignment	Abd-B Peptide	*Cloeon dipterum*	CAB3384558.1	NCBI
Phylogeny	Peptide Database	*Hexagenia limbata*	N/A	[1]
Phylogeny	Genome	*Orchesella cincta*	GCA_001718145.1	NCBI
Phylogeny	Genome	*Folsomia candida*	GCF_002217175.1	NCBI
Phylogeny	Genome	*Ladona fulva*	GCA_000376725.2	NCBI
Phylogeny	Genome	*Blattella germanica*	GCA_003018175.1	NCBI
Phylogeny	Genome	*Zootermopsis nevadensis*	GCF_000696155.1	NCBI
Phylogeny	Genome	*Diuraphis noxia*	GCF_001186385.1	NCBI
Phylogeny	Genome	*Thrips palmi*	GCF_012932325.1	NCBI
Phylogeny	Genome	*Frankliniella occidentalis*	GCF_000697945.2	NCBI
Phylogeny	Genome	*Apis mellifera*	GCF_003254395.2	NCBI
Phylogeny	Genome	*Tribolium castaneum*	GCF_000002335.3	NCBI
Phylogeny	Genome	*Chrysoperla carnea*	GCF_905475395.1	NCBI
Phylogeny	Genome	*Bombyx mori*	GCF_014905235.1	NCBI
Phylogeny	Genome	*Drosophila melanogaster*	GCF_000001215.4	NCBI
Hox cluster	Chromosome	*Drosophila melanogaster*	NT_033777.3	NCBI
Hox cluster	Chromosome	*Bombyx mori*	NC_051363.1	NCBI
Hox cluster	Chromosome	*Tribolium castaneum*	NC_007417.3	NCBI
Hox cluster	Scaffold	*Ephemera danica*	KZ497623.1	NCBI
Hox cluster	Scaffold	*Ephemera danica*	KZ497756.1	NCBI
Hox cluster	Scaffold	*Folsomia candida*	NW_019091196.1	NCBI

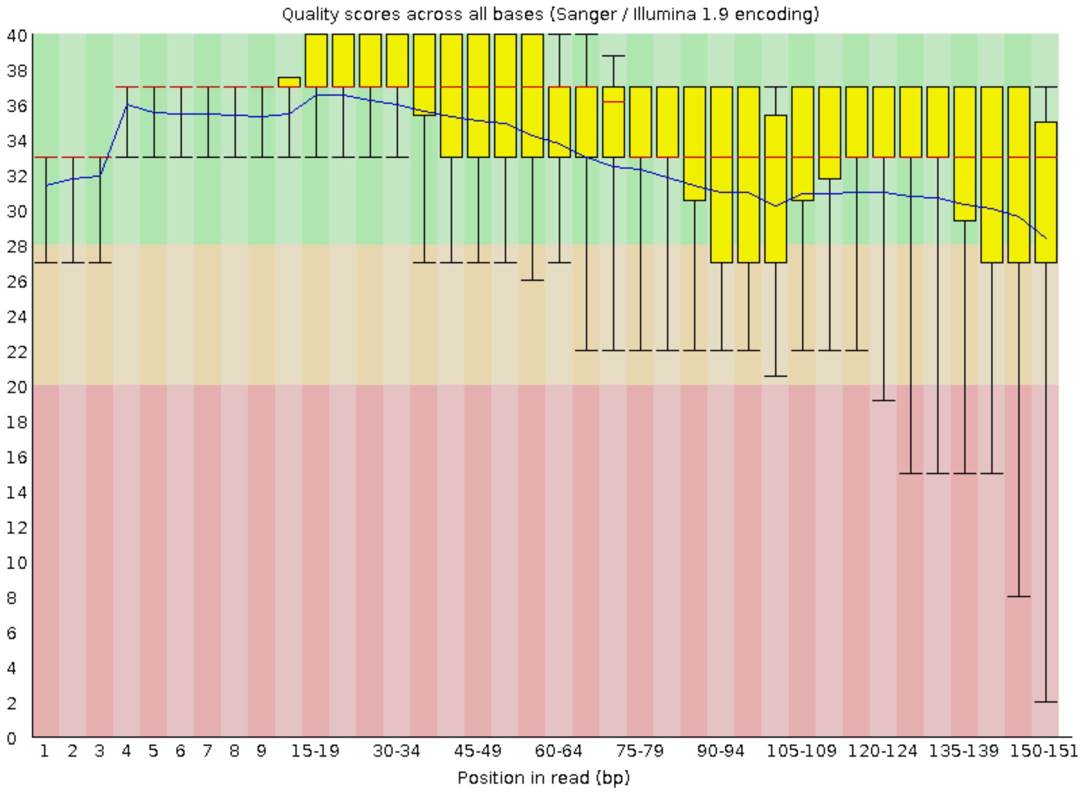

Additional file 1: Fig. S3. Box plot of R1 forward read Phred quality scores across all base pairs, as provided by FastQC. Yellow boxes denote the 25%-75% inter-quartile range, with the 10% and 90% range shown via whiskers. Mean and median quality scores are provided by the blue and red lines, respectively.

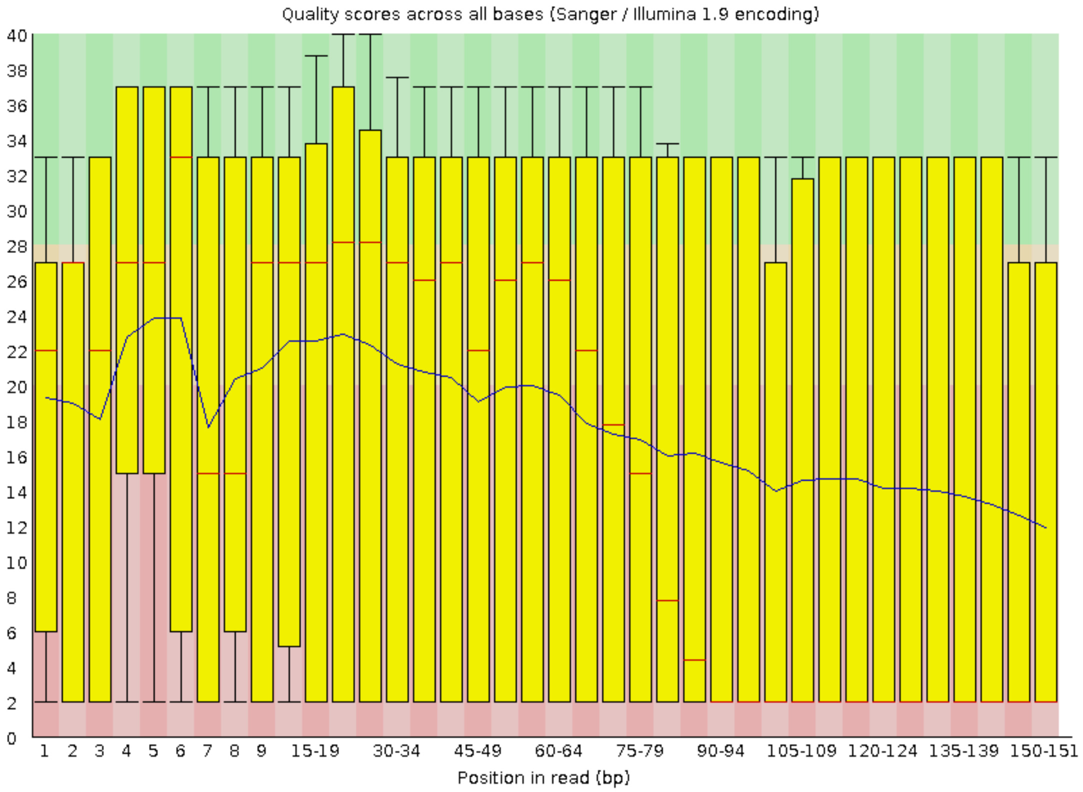

Additional file 1: Fig. S4. Box plot of R3 reverse read Phred quality scores across all base pairs, as provided by FastQC. Yellow boxes denote the 25%-75% inter-quartile range, with the 10% and 90% range shown via whiskers. Mean and median quality scores are provided by the blue and red lines, respectively.

Additional File 1: Table S2 Top three reciprocal BLASTp hits for putative *H. limbata* Hox proteins.

peptide	Species	Abbreviated Description	Version	E-Value
Lab	*Nilaparvata lugens*	Hox-B1a-like	XP_022198853.1	4E-59
	Aethina tumida	PREDICTED: Hox-B1b-like	XP_019875930.1	3E-53
	Agrilus planipennis	Hox-A1-like	XP_018322094.1	3E-53
Pb	*Cephus cinctus*	proboscipedia	XP_015600387.1	4E-90
	Formica exsecta	proboscipedia	XP_029663337.1	1E-85
	Chelonus insularis	proboscipedia	XP_034949502.1	3E-83
Dfd	*Cimex lectularius*	Hox-B4 isoform X2	XP_024083281.1	1E-75
	Cimex lectularius	Hox-B4 isoform X3	XP_014240109.1	3E-75
	Cryptotermes secundus	deformed	XP_023702719.1	1E-74
Scr	*Diabrotica*	Sex combs reduced-like isoform X1	XP_028132252.1	5E-98
	Nicrophorus vespilloides	PREDICTED: Sex combs reduced-like	XP_017768265.1	3E-97
	Cryptotermes secundus	Sex combs reduced	XP_023702514.1	2E-96

Antp	*Onthophagus taurus*	antennapedia	XP_022907641.1	2E-93
	Nicrophorus	PREDICTED: antennapedia-like	XP_017772124.1	2E-91
	Sitophilus oryzae	antennapedia-like	XP_030758247.1	2E-90
Ubx	*Thrips palmi*	ultrabithorax isoform X2	XP_034234417.1	2E-104
	Bemisia tabaci	PREDICTED: ultrabithorax	XP_018908365.1	4E-102
	Orchesella cincta	Ultrabithorax	CDI44541.1	6E-102
Abd-A	*Fopius arisanus*	PREDICTED: abd-A homolog isoform X1	XP_011311715.1	2E-103
	Microplitis demolitor	PREDICTED: abd-A homolog isoform X1	XP_008546318.1	3E-103
	Vollenhovia emeryi	PREDICTED: abd-A homolog isoform X1	XP_011870678.1	5E-103
Abd-B	*Parhyale hawaiensis*	abdominal-B isoform I	AGC12523.1	1E-93
	Hyalella azteca	PREDICTED: abdominal-B-like	XP_018009927.1	1E-91
	Parhyale hawaiensis	abdominal-B isoform II	AGC12524.1	3E-91

H. limbata Hox proteins are the same used for alignments in Additional files 2: S12-19. *H. limbata* accession values are reported in Additional file 1: Table S1.

Additional file 1: Table S3 Top three reciprocal BLASTp hits for putative *E. danica* Hox proteins.

peptide	Species	Abbreviated Description	Version	E-Value
Lab	*Ailuropoda melanoleuca*	Hox-B3	XP_034507441.1	6E-31
	Myotis brandtii	PREDICTED: Hox-B3	XP_014396619.1	1E-28
	Ailuropoda melanoleuca	Hox-B3	XP_034496124.1	2E-28
Pb	*Nomia melanderi*	proboscipedia	XP_031846347.1	1E-64
	Chelonus insularis	proboscipedia	XP_034949502.1	5E-64
	Halyomorpha halys	proboscipedia	XP_014283953.1	8E-64
Zen	*Ailuropoda melanoleuca*	Hox-B3	XP_034507441.1	6E-31
	Myotis brandtii	PREDICTED: Hox-B3	XP_014396619.1	1E-28
	Ailuropoda melanoleuca	Hox-B3	XP_034496124.1	2E-28
Dfd	*Asbolus verrucosus*	transcription factor deformed	RZC40226.1	7E-66
	Halyomorpha halys	Hox-A4 isoform X2	XP_014283974.1	3E-65
	Cryptotermes secundus	deformed	XP_023702719.1	8E-65
Ftz	*Limulus polyphemus*	Hox-A7-like isoform X2	XP_013776722.2	3E-42
	Phalangium opilio	fushi tarazu	CCH51005.1	2E-40
	Photinus pyralis	Hox-A10-like	XP_031358951.1	2E-40
Scr	*Tribolium castaneum*	PREDICTED: cephalothorax isoform X1	XP_008201245.1	4E-56
	Nicrophorus vespilloides	PREDICTED: Sex combs reduced-like	XP_017768265.1	7E-56
	Dendroctonus ponderosae	PREDICTED: Sex combs reduced isoform X1	XP_019760916.1	8E-56
Antp	*Onthophagus taurus*	antennapedia	XP_022907641.1	8E-93
	Nicrophorus vespilloides	PREDICTED: antennapedia-like	XP_017772124.1	1E-90
	Cryptotermes secundus	antennapedia	XP_023702421.1	5E-90
Ubx	*Polistes canadensis*	PREDICTED: ultrabithorax-like	XP_014615332.1	2E-58
	Amyelois transitella	PREDICTED: ultrabithorax-like	XP_013193930.1	2E-58
	Drosophila sechellia	GM15411	EDW44822.1	6E-58
Abd-A	*Formica exsecta*	abdominal-A homolog	XP_029663382.1	2E-57
	Trachymyrmex cornetzi	abdominal-A like protein	KYN13903.1	2E-57
	Agrilus planipennis	abdominal-A homolog	XP_025828928.1	2E-57
Abd-B	*Parhyale hawaiensis*	abdominal-B isoform I	AGC12523.1	1E-96
	Hyalella azteca	PREDICTED: abdominal-B-like	XP_018009927.1	4E-95
	Parhyale hawaiensis	abdominal-B isoform II	AGC12524.1	3E-94

Top hits exclude any hypothetical proteins. *E. danica* Hox peptides are the same used for alignments in Additional file 1: S9-S10 and S12-S19. *E. danica* accession values are reported in Additional file 1: Table S1.

Additional file 1: Table S4 Top three reciprocal BLASTp hits for putative C. *dipterum* Hox proteins.

peptide	Species	Abbreviated Description	Version	E-Value
Lab	*Anoplophora glabripennis*	Hox-A1	XP_018562503.1	8E-53
	Agrilus planipennis	Hox-A1-like	XP_018322094.1	3E-52
	Photinus pyralis	Hox-A1-like	XP_031338018.1	4E-52
Pb	*Hyposmocoma kahamanoa*	proboscipedia	XP_026330163.1	8E-73
	Vanessa tameamea	proboscipedia	XP_026485409.1	4E-69
	Amyelois transitella	PREDICTED: proboscipedia	XP_013193968.1	4E-69
Dfd	*Bemisia tabaci*	PREDICTED: Hox-B4	XP_018902413.1	2E-69
	Cimex lectularius	Hox-B4 isoform X3	XP_014240109.1	3E-69
	Photinus pyralis	Hox-C4-like isoform X2	XP_031341519.1	6E-68
Scr	*Nicrophorus vespilloides*	PREDICTED: Sex combs reduced-like	XP_017768265.1	2E-88
	Diabrotica virgifera virgifera	Sex combs reduced-like isoform X1	XP_028132252.1	8E-86
	Tribolium castaneum	PREDICTED: cephalothorax isoform X1	XP_008201245.1	3E-85
Antp	*Onthophagus taurus*	antennapedia	XP_022907641.1	8E-97
	Photinus pyralis	antennapedia-like isoform X2	XP_031337143.1	5E-92
	Anoplophora glabripennis	antennapedia-like	XP_018561276.1	6E-91
Ubx	*Orchesella cincta*	Ultrabithorax	CDI44537.1	3E-98
	Orchesella cincta	Ultrabithorax	CDI44538.1	9E-97
	Orchesella cincta	Ultrabithorax	CDI44541.1	1E-96
Abd-A	*Halyomorpha halys*	abdominal-A homolog	XP_014274749.1	2E-103
	Cimex lectularius	abdominal-A homolog	XP_014240425.1	2E-100
	Agrilus planipennis	abdominal-A homolog	XP_025828944.1	3E-100
Abd-B	*Strigamia maritima*	abdominal-B	ABD16214.1	2E-60
	Amphibalanus amphitrite	abdominal-B	KAF0297071.1	2E-60
	Hyalella azteca	PREDICTED: abdominal-B-like	XP_018009927.1	6E-60

Top hits exclude any hypothetical proteins. C. *dipterum* Hox peptides are the same used for alignments in Additional file 1: S5-S8 and S11-S19. C. *dipterum* accession values are reported in Additional file 1: Table S1.

REFERENCES

1 1. Gonzalez, Christopher, Hildebrandt, Tobias, O'Donnell, Brigid. *Hexagenia limbata* early instar transcriptome assemblies. Zenodo; 2022. https://doi.org/10.5281/zenodo.6624929
