# Supplementary material for: Characterizing Hox genes in mayflies (Ephemeroptera), with Hexagenia limbata as a new mayfly model
Source: EvoDevo. 2022 Jul 27;13:15. doi: 10.1186/s13227-022-00200-w (PMC9331126; doi:10.1186/s13227-022-00200-w)
Supplement: Supplementary file 2 — Additional file 2. Contains all alignments generated in this study, including assessments of the mayfly Hox scaffolds and all identified mayfly Hox proteins. [file 13227_2022_200_MOESM2_ESM.pdf]

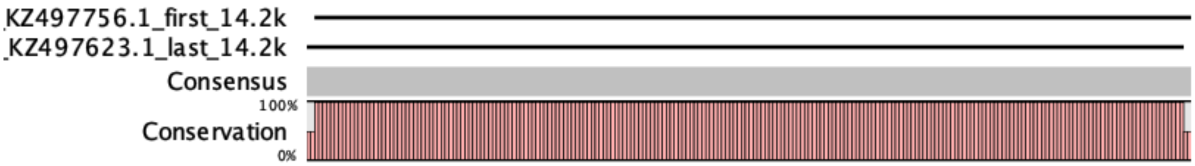

Additional file 2: Fig. S1 Partial alignment of the two *E. danica* scaffolds, KZ497623.1 and KZ497756.1, which contain the putative *E. danica* Hox homologs. Alignment contains the last 14,200bp of KZ497623.1, and the first 14,200bp of KZ497756.1. The overlapping region entails the last 14077bp of KZ497623.1 and the first 14,081bp of KZ497756.1, creating a 14085bp overlap that is over 99.8% identical. The consensus sequence for this overlap was thus used to concatenate these scaffolds.

```
     1                                                          60
     |         |         |         |         |         |         |
ORP agtggtatcagtgagtaatggtggatctctagatagacttagtcctccacccccagaatc
CLC ------------------------------------------------------------

              70                                               120
               |         |         |         |         |         |
ORP cccaagtaccacagtagatcaacattcaaatcttggagattatgttggggatgaatctgg
CLC ------------------------------------------------------------

              130                                              180
               |         |         |         |         |         |
ORP acctgatgaggatgaagatgattcaggatcagatcaaggcagcgatcgagttatttatcc
CLC ----------------------------------------------------atttatcc
                                                        ********

              190                                              240
               |         |         |         |         |         |
ORP ttggatgcgtaaaatccatgtggctggagttggtgagtgtcccacgtctatggcaaacgg
CLC ttggatgcgtaaaatccatgtggctggagttggtgagtgtcccacgtctatggcaaacgg
    ************************************************************

              250                                              300
               |         |         |         |         |         |
ORP ttcatttcaaccaggtatggaaccaaaacggcaaagaacagcttatacgagacatcaaat
CLC ttcatttcaaccaggtatggaaccaaaacggcaaagaacagcttatacgagacatcaaat
    ************************************************************

              310                                              360
               |         |         |         |         |         |
ORP acttgaattagaaaaagaatttcattttaatcgttatttaacaaggcgaagacgtatcga
CLC acttgaattagaaaaagaatttcattttaatcgttatttaacaaggcgaagacgtatcga
    ************************************************************

              370                                              420
               |         |         |         |         |         |
ORP aattgctcattcattatgtttatctgaaagacagattaagatttggtttcaaaatcgtcg
CLC aattgctcattcattatgtttatctgaaagacagattaagatttggtttcaaaatcgtcg
    ************************************************************

              430                                              480
               |         |         |         |         |         |
ORP tatgaaatggaaaaaagataataaattaccaaatactaaaaatgtaagaa----------
CLC tatgaaatggaaaaaagataataaattaccaaatactaaaaatgtaagaagaaaaacaaa
    **************************************************
```

```
            490                                               540
             |         |         |         |         |         |
ORP ------------------------------------------------------------
CLC tcctgctggtgttactactaccaaaccacgaggtaaaagtcgagctgctgctaatcgtaa

            550                                               600
             |         |         |         |         |         |
ORP ------------------------------------------------------------
CLC taataacaataataacagtaacaatattgaaaatactagccttggtacgaatggaccatt

            610                                               660
             |         |         |         |         |         |
ORP ------------------------------------------------------------
CLC atctcaacttggttccatgcatcaaggactacaggaaagtacaggtactccaccaccttt

            670                                               720
             |         |         |         |         |         |
ORP ------------------------------------------------------------
CLC gcacttatcacctgcgtctttaagtcaacaattaacacctttaactcctcaaacttgtgg

            730                                     770
             |         |         |         |         |
ORP -------------------------------------------------------
CLC tcctaatcatccacccccgcctatgaagtcagattatggacttactgctctctga
```

Additional file 2: Fig. S2 cDNA alignment of open reading frames from CLC contig 55031 and ORP contig NODE_44332_length_470_cov_1.669880_g34783_i0 to form the consensus *H. limbata* Deformed (Dfd) sequence. Concatenation of these sequences, as shown translated in Additional file 2: Fig. S14, is justified by the 100% identical overlap (blue). Contig databases are provided in Additional file 1: Table S1.

```
  SSYF
  1                                                          60
  |             |             |             |             |             |
Ubx MNSYFEQTGFYGSQSTAEQPYRFPLGLGVGMSPYAQHHHQSRPPQDSPYDATAAAAAAAA
Alt MNSYFEQTGFYGSQSTAEQPYRFPLGLGVGMSPYAQHHHQSRPPQDSPYDATAAAAAAAA
    ************************************************************

             70                                                 120
             |             |             |             |             |
Ubx AAAASCKLYSSQMTADSYKLDCTTKNSASNNNTSNSNEHHSNGYSSVSAAAALAAATASN
Alt AAAASCKLYSSQMTADSYKLDCTTKNSASNNNTSNSNEHHSNGYSSVSAAAALAAATASN
    ************************************************************

             130                                                180
             |             |             |             |             |
Ubx KSDIASWQNAASSGGGVPVRPAACTPDGAVSRYSGTGNGSLSGADPSVTTSPSSAVAAAH
Alt KSDIASWQNAASSGGGVPVRPAACTPDGAVSRYSGTGNGSLSGADPSVTTSPSSAVAAAH
    ************************************************************
                                                Hx
             190                                                240
             |             |             |             |             |
Ubx ARSLGGSWNTCSLNTSAAAVQQQAVQQPANHTFYPWMAIAGANGLRRRGRQTYTRYQTLE
Alt ARSLGGSWNTCSLNTSAAAVQQQAVQQPANHTFYPWMAIAGANGLRRRGRQTYTRYQTLE
    ************************************************************
                          HD                             UbdA   QAQA
             250                                                300
             |             |             |             |             |
Ubx LEKEFHTNHYLTRRRRIEMAHALCLTERQIKIWFQNRRMKLKKEIQAIKELNEQEKQAQA
Alt LEKEFHTNHYLTRRRRIEMAHALCLTERQIKIWFQNRRMKLKKEIQAIKELNEQEKQAQA
    ************************************************************
       Poly-A
             310
             |
Ubx QKAAAAAAAAQASQGGGD
Alt RKAAAAAAAAQASQGGGD
    :*****************
```

Additional file 2: Fig. S3 Amino acid alignment of putative *H. limbata* Ubx with an alternative Ubx sequence identified in the ORP assembly. Alt replaces Ubx in Fig. 1; Ubx is reported in BLAST results and Additional file 2: Fig. S17. Note that all residues are identical except for residue 301. Sequence annotations marked in bold, as follows: SSYF motif (red); Hx, Hexapeptide motif (green); Homeodomain (blue); UbdA motif (orange); Poly-A region (grey). All sequence accession numbers are reported in Additional file 1: Table S1.

```
        SSYF
        1                                                          60
        |         |         |         |         |         |         |
04961   MTSKFIDSMLPKYQSETASSVVNYSQSAAAQHHARMYPYVSMGTPHQQAAAAAAFGAAAA
Abd-A   MTSKFIDSMLPKYQSETASSVVNYSQSAAAQHHARMYPYVSMGTPHQQAAAAAAFGAAAA
        ************************************************************

                 70                                                120
                  |         |         |         |         |         |
04961   ASSTMVPFSSSLAAAAASAGDAGDKSCRYSNPATGLSADSMVNYTLSHAAAAQNGASTTS
Abd-A   ASSTMVPFSSSLAAAAASAGDAGDKSCRYSNPATGLSADSMVNYTLSHAAAAQNGASTTS
        ************************************************************
                                                     Hx       TDWM       PFER
                130                                                180
                  |         |         |         |         |         |
04961   TMAAAAQFYHQAAAASDPSNPLTSCSQTPGQPIPDIPRYPWMSITDSLFGSLDWMSPFDR
Abd-A   TMAAAAQFYHQAAAASDPSNPLTSCSQTPGQPIPDIPRYPWMSIT-------DWMSPFDR
        *********************************************       ********
                                                        HD
                190                                                240
                  |         |         |         |         |         |
04961   VVCDMSFVTGPNGCPRRRGRQTYTRFQTLELEKEFHFNHYLTRRRRIEIAHALCLTERQI
Abd-A   VVCDMSFVTGPNGCPRRRGRQTYTRFQTLELEKEFHFNHYLTRRRRIEIAHALCLTERQI
        ************************************************************
                              UbdA
                250                                                300
                  |         |         |         |         |         |
04961   KIWFQNRRMKLKKELRAVKEINEQARREREE-----------------------------
Abd-A   KIWFQNRRMKLKKELRAVKEINEQARREREEQERLKQQQQQQQDKAAKQHAEQLQQATKL
        *******************************

                310
                  |
04961   --------------------
Abd-A   AMDKALIGGDLIKGKYIDCI
```

Additional file 2: Fig. S4 Amino acid alignment of putative *H. limbata* Abd-A with an additional Abd-A sequence identified in the ORP assembly. Both proteins are depicted in Fig. 1; Abd-A is reported in BLAST results and Additional file 2: Fig. S18. Sequence annotations marked in bold, as follows: SSYF motif (red); Hx, Hexapeptide motif (green); TDWM motif (yellow); PFER motif (light blue); HD, Homeodomain (blue); UbdA motif (orange). All sequence accession numbers are reported in Additional file 1: Table S1.

```
      1                                                         60
      |        |         |         |         |         |         |
Pb    MPALPCEPKSTGVGQKAAKRRPGPQLLLEADHVGMRPAARALCGAPNVKVSGKLNERAPL
10066 MPALPCEPKSTGVGQKAAKRRPGPQLLLEADHVGMRPAARALCGAPNVKVSGKLNERAPL
10068 M-----------------------------------------------------------
      *

              70                                               120
      |        |         |         |         |         |         |
Pb    REDYLQAVAAAVDNCWGPLGAAMDHCNETGFINSEPSMAEYISRIPDPRESQSPFLEVAT
10066 REDYLQAVAAAVDNCWGPLGAAMDHCNETGFINSEPSMAEYISRIPDPRESQSPFLEVAT
10068 -------------------------CLELGIM----------------------------
                               * * *::
                             Hx

             130                                               180
      |        |         |         |         |         |         |
Pb    AHADGRGLMQQTAAVSAASVPEYPWMKEKKTSRKNSTPE---------------------
10066 AHADGRGLMQQTAAVSAASVPEYPWMKEKKTSRKNSTPELRGIFMDSEAENEPGDHGRGS
10068 ----------------------FPEMVENPPDLQ----E---------------------
                            :* * *: .. :    *
                                           HD

             190                                               240
      |        |         |         |         |         |         |
Pb    --------NGLPRRLRTAYTNTQLLELEKEFHFNKYLCRPRRIEIAASLDLTERQVKVWF
10066 TGRTNRHKNGLPRRLRTAYTNTQLLELEKEFHFNKYLCRPRRIEIAASLDLTERQVKVWF
10068 --------NGLPRRLRTAYTNTQLLELEKEFHFNKYLCRPRRIEIAASLDLTERQVKVWF
              ****************************************************

             250                                               300
      |        |         |         |         |         |         |
Pb    QNRRMKHKRQTVGKGEDDKESSKEKAKEDKKSCQNCELPGGPLTSGNNNNNSAKNNNSSN
10066 QNRRMKHKRQTVGKGEDDKESSKEKAKEDKKSCQNCELPGGPLTSGNNNNNSAKNNNSSN
10068 QNRRMKHKRQTVGKGEDDKESSKEKAKEDKKSCQNCELPGGPLTSGNNNNNSAKNNNSSN
      ************************************************************

             310                                               360
      |        |         |         |         |         |         |
Pb    TFSSSSSTSSSFKEEDSQSRESGVLTPSIKMADVIKMEVKHSPIDASPKTPPVEALAEPS
10066 TFSSSSSTSSSFKEEDSQSRESGVLTPSIKMADVIKMEVKHSPIDASPKTPPVEALAEPS
10068 TFSSSSSTSSSFKEEDSQSRESGVLTPSIKMADVIKMEVKHSPIDASPKTPPVEALAEPS
      ************************************************************

             370                                               420
      |        |         |         |         |         |         |
Pb    LGSLTPSTSSPPHPSPAAYRHPSPSPAALQATPAPTVAVGVRRNFKNTPYRTDYPSRAYT
10066 LGSLTPSTSSPPHPSPAAYRHPSPSPAALQATPAPTVAVGVRRNFKNTPYRTDYPSRAYT
10068 LGSLTPSTSSPPHPSPAAYRHPSPSPAALQATPAPTVAVGVRRNFKNTPYRTDYPSRAYT
      ************************************************************

             430                                               480
      |        |         |         |         |         |         |
Pb    ASQGRFPGQPTQIRHQYTSSSIQNDYRRTNGLHNPVTTPRTQQMQSSNRYYQGYNFYYQP
10066 ASQGRFPGQPTQIRHQYTSSSIQNDYRRTNGLHNPVTTPRTQQMQSSNRYYQGYNFYYQP
10068 ASQGRFPGQPTQIRHQYTSSSIQNDYRRTNGLHNPVTTPRTQQMQSSNRYYQGYNFYYQP
      ************************************************************

             490                                               540
      |        |         |         |         |         |         |
Pb    QQQQQQQQDGTATYYTQAQLQQQQQQQQQHQRTYQAQYEEGYHNQQQVQGNGPYYNPNSG
10066 QQQQQQQQDGTATYYTQAQLQQQQQQQQQHQRTYQAQYEEGYHNQQQVQGNGPYYNPNSG
10068 QQQQQQQQDGTATYYTQAQLQQQQQQQQQHQRTYQAQYEEGYHNQQQVQGNGPYYNPNSG
      ************************************************************

             550                                               600
      |        |         |         |         |         |         |
Pb    YYAQHQQQQQPQVSGEAIGQFYDNAGNYNAHHQQQYQKGSTYYSAADLMQGGEASNSHYV
10066 YYAQHQQQQQPQVSGEAIGQFYDNAGNYNAHHQQQYQKGSTYYSAADLMQGGEASNSHYV
10068 YYAQHQQQQQPQVSGEAIGQFYDNAGNYNAHHQQQYQKGSTYYSAADLMQGGEASNSHYV
      ************************************************************
```

```
              610                                                660
                |        |        |        |        |        |
Pb    SSPDTFPAAATATAVSTSLQPQQQQLPAQATEHAQNFVQFPAATNAFYEQAGASTSTNPP
10066 SSPDTFPAAATATAVSTSLQPQQQQLPAQATEHAQNFVQFPAATNAFYEQAGASTSTNPP
10068 SSPDTFPAAATATAVSTSLQPQQQQLPAQATEHAQNFVQFPAATNAFYEQAGASTSTNPP
      ************************************************************

              670                      700
                |        |        |        |
Pb    VVAAPAVAASSNSMASENSNSSSEFNFFSTLANDFAPEYYQLS
10066 VVAAPAVAASSNSMASENSNSSSEFNFFSTLANDFAPEYYQLS
10068 VVAAPAVAASSNSMASENSNSSSEFNFFSTLANDFAPEYYQLS
      *******************************************
```

Additional file 2: Fig. S5 Amino acid alignment of putative *C. dipterum* Pb with additional Pb sequences. All sequences are depicted in Fig.1; Pb is reported in BLAST results and Additional file 2: Fig. S13. Sequence annotations marked in bold, as follows: Hx, Hexapeptide motif (green); HD, Homeodomain (blue). All sequence accession numbers are reported in Additional file 1: Table S1.

```
        SSYF
      1                                                         60
      |        |         |         |         |         |
10062 MQQDASAMNSVYLNSYPAAAADPKFPPPEADYGQGPFMSATPDYFHQNSYAEHYVAPVPA
Alt   MQQDASAMNSVYLNSYPAAAADPKFPPPEADYGQGPFMSATPDYFHQNSYAEHYVAPVPA
Dfd   MQQDASAMNSVYLNSYPAAAADPKFPPPEADYGQGPFMSATPDYFHQNSYAEHYVAPVPA
      ************************************************************

              70                                               120
               |         |         |         |         |         |
10062 GAAPPAPYQQYHHHHQSPYYPIHAAPQQHLYHVPPAAPAPPHLAPTVLSGGGGGDASPHV
Alt   GAAPPAPYQQYHHHHQSPYYPIHAAPQQHLYHVPPAAPAPPHLAPTVLSGGGGGDASPHV
Dfd   GAAPPAPYQQYHHHHQSPYYPIHAAPQQHLYHVPPAAPAPPHLAPTVLSGGGGGDASPHV
      ************************************************************
                                                      Hx
             130                                               180
               |         |         |         |         |         |
10062 VQHASPAESPSTTADQHSMLDYPGDDSSGEMEDDDEESGDDGGDRVIYPWMKKIHVAGVA
Alt   VQHASPAESPSTTADQHSMLDYPGDDSSGEMEDDDEESGDDGGDRVIYPWMKKIHVAGVG
Dfd   VQHASPAESPSTTADQHSMLDYPGDDSSGEMEDDDEESGDDGGDRVIYPWMKKIHVAGVA
      ***********************************************************.
                                             HD
             190                                               240
               |         |         |         |         |         |
10062 ----IGQFQPGMEPKRQRTAYTRHQILELEKEFHFNRYLTRRRRIEIAHSLNLSERQIKI
Alt   KPRTIGQFQPGMEPKRQRTAYTRHQILELEKEFHFNRYLTRRRRIEIAHSLNLSERQIKI
Dfd   ----IGQFQPGMEPKRQRTAYTRHQILELEKEFHFNRYLTRRRRIEIAHSLNLSERQIKI
          ********************************************************

             250                                               300
               |         |         |         |         |         |
10062 WFQNRRMKWKKDNKLPNTKNVRRKNANGQPSPPKSRAKRGRSNNNVDGSSNLNDPLSQLG
Alt   WFQNRRMKWKKDNKLPNTKNVRRKNANGQPSPPKSRAKRGRSNNNVDGSSNLNDPLSQLG
Dfd   WFQNRRMKWKKDNKLPNTKNVRRKNANGQPSPPKSRAKRGRSNNNVDGSSNLNDPLSQLG
      ************************************************************

             310                                     350
               |         |         |         |         |
10062 AMNPGDRQPQLHLSSATIDHQ--------CHLS------DWTLCGHVDVILVRGCRV
Alt   AMNPGDRQPQLHLSSATIDHQVTPLTPQSCHLQPPPLKQDFNL-----------TPL
Dfd   AMNPGDRQPQLHLSSATIDHQVTPLTPQSCHLQPPPLKQDFNL-----------TPL
      *********************        ***.      *:.*             :
```

Additional file 2: Fig. S6 Amino acid alignment of putative *C. dipterum* Dfd with additional Dfd sequences. The alternative sequence and 10062 are depicted in Fig.1; Dfd is reported in BLAST results and Additional file 2: Fig. S14. Sequence annotations marked in bold, as follows: SSYF motif (red); Hx, Hexapeptide motif (green); HD, Homeodomain (blue). All sequence accession numbers are reported in Additional file 1: Table S1.

```
        SSYF
     1                                                          60
     |             |              |             |             |             |
Antp MSSYFANSYIPDLRNGTPDHYATTAHQGAQVNSDPSCDPSLRQGIPNHYGGPPAAGQPPP
Alt  MSSYFANSYIPDLRNGTPDHYATTAHQGAQVNSDPSCDPSLRQGIPNHYGGPPAAGQPPP
     ************************************************************

              70                                                120
              |             |             |             |             |
Antp GMPYPRFPPYDRLDIRAAGYYGTQTGQTAQNGAVGLLVDNTNAGYRPNSPTMGGAHMPQQ
Alt  GMPYPRFPPYDRLDIRAAGYYGTQTGQTAQNGAVGLLVDNTNAGYRPNSPTMGGAHMPQQ
     ************************************************************

             130                                                180
              |             |             |             |             |
Antp TTPVQYSSCKLQANAAHMGQHHMAQQAHMVQYNNVAQQPQQIPHQVHQPPHNNQLHPPQ
Alt  TTPVQYSSCKLQANAAHMGQHHMAQQAHMVQYNNVAQQPQQIPHQVHQPPHNNQLHPPQ
     ************************************************************
                         Hx                            HD
             190                                                240
              |             |             |             |             |
Antp QHTPPQQQTNNSSANLPSPLYPWMRSQ---FERKRGRQTYTRYQTLELEKEFHFNRYLTR
Alt  QHTPPQQQTNNSSANLPSPLYPWMRSQFGKFERKRGRQTYTRYQTLELEKEFHFNRYLTR
     ***************************   ******************************

             250                                      290
              |             |             |             |
Antp RRRIEIAHALCLTERQIKIWFQNRRMKWKKENKSKSDSGSQHDGMDATPPDSPQ
Alt  RRRIEIAHALCLTERQIKIWFQNRRMKWKKENKSKSDSGSQHDGMDATPPDSPQ
     ******************************************************
```

Additional file 2: Fig. S7 Amino acid alignment of putative *C. dipterum* Antp with an alternative Antp sequence. The alternative sequence replaces Antp in Fig.1; Antp is reported in BLAST results and Additional file 2: Fig. S16. Sequence annotations marked in bold, as follows: SSYF motif (red); Hx, Hexapeptide motif (green); HD, Homeodomain (blue). All sequence accession numbers are reported in Additional file 1: Table S1.

```
        SSYF
        1                                                          60
        |         |         |         |         |         |         |
Alt    MTSKFIDTMLPKYSGQDTSSYTQSASHHARMYPYVSMGTHQQAAAAAAAFNAAAAAASSTM
07337  MTSKFIDTMLPKYSGQDTSSYTQSASHHARMYPYVSMGTHQQAAAAAAAFNAAAAAASSTM
Abd-A  MTSKFIDTMLPKYSGQDTSSYTQSASHHARMYPYVSMGTHQQAAAAAAAFNAAAAAASSTM
       ************************************************************

                70                                              120
                |         |         |         |         |         |
Alt    VPFSTSLAAAAASVSDSDKSCRYSNPATGLSAADSMVNYTLGHQNGSAAPSSMAAAAQFY
07337  VPFSTSLAAAAASVSDSDKSCRYSNPATGLSAADSMVNYTLGHQNGSAAPSSMAAAAQFY
Abd-A  VPFSTSLAAAAASVSDSDKSCRYSNPATGLSAADSMVNYTLGHQNGSAAPSSMAAAAQFY
       ************************************************************
                                       Hx       TDWM      PFER
               130                                              180
                |         |         |         |         |         |
Alt    HQTAAAASDPANPLTTACTQASGQPLPDIPRYPWMSITDSLFGSLDWMSPFDRVVCGPYD
07337  HQTAAAASDPANPLTTACTQASGQPLPDIPRYPWMSIT-------DWMSPFDRVVCG---
Abd-A  HQTAAAASDPANPLTTACTQASGQPLPDIPRYPWMSIT-------DWMSPFDRVVCGPYD
       **************************************       ************
                                           HD
               190                                              240
                |         |         |         |         |         |
Alt    GLGSPNGCPRRRGRQTYTRFQTLELEKEFHFNHYLTRRRRIEIAHALCLTERQIKIWFQN
07337  ----PNGCPRRRGRQTYTRFQTLELEKEFHFNHYLTRRRRIEIAHALCLTERQIKIWFQN
Abd-A  GLGSPNGCPRRRGRQTYTRFQTLELEKEFHFNHYLTRRRRIEIAHALCLTERQIKIWFQN
           ********************************************************
                     UbdA
               250                                              300
                |         |         |         |         |         |
Alt    RRMKLKKELRAVKEINEQARREREEQERIKSQQQQQHEKAAKQHAEQLHQATKLAMDKAL
07337  RRMKLKKELRAVKEINEQARREREEQERIKSQQQQQHEKAAKQHAEQLHQATKLAMDKAL
Abd-A  RRMKLKKELRAVKEINEQARREREEQERIKSQQQQQHEKAAKQHAEQLHQATKLAMDKAL
       ************************************************************

               310
                |
Alt    MGGDLIKGVGKAS
07337  MGGDLIKGVGKAS
Abd-A  MGGDLIKGVGKAS
       *************
```

Additional file 2: Fig. S8 Amino acid alignment of putative *C. dipterum* Abd-A with additional Abd-A sequences. The alternative sequence and 07337 are depicted in Fig.1; Abd-A is reported in BLAST results and Additional file 2: Fig. S18. Sequence annotations marked in bold, as follows: SSYF motif (red); Hx, Hexapeptide motif (green); TDWM motif (yellow); PFER motif (light blue); HD, Homeodomain (blue); UbdA motif (orange). All sequence accession numbers are reported in Additional file 1: Table S1.

```
                                                  HD

     1                                                          60
     |        |         |         |         |         |         |
D.m. -------------SQPNHDSQRVKLKRSRTAFTSVQLVELENEFKSNMYLYRTRRIEIAQ
T.c. -------TVTEIQETQPQRTKPTAGKRARTAYTSAQLVELEREFHHGKYLSRPRRIQIAE
F.o. ---------SSTTSSSSSSSSSSTSKRARTAYTSAQLVELEKEFHFNRYLCRPRRIEMAA
F.c. PLLHDQVKSCQPTSSSSTEGGDHPTKRARTAYTSAQLVELEKEFHYNRYLCRPRRIEMAS
E.d. ------CGPHGINNNSPRPVQ--QAKRARTAYTSAQLVELEKEFSMNRYLCRPRRIELAT
                              ** *** ** ****** **    ** * ***  *
                                                       C-terminus
             70                                                120
              |         |         |         |         |         |
D.m. RLSLCERQVKIWFQNRRMKFKKDIQ---------------------GHREPKSNAKLAQP
T.c. NLNLSERQIKIWFQNRRMKHKKE--------QMNK---------------------VSTP
F.o. LLSLTERQIKIWFQNRRMKYKKE--------QRGR-----------GPGDESTKTAPGKP
F.c. LLSLTERQIKIWFQNRRMKYKKELKSPNSSSQGGKSGGGGGGDCCGGSGSPPPPLKQGSP
E.d. SLCLSERQIKIWYQNRRMKLKKE--------QRLK-----------QQEETKSTASASPP
      * * *** *** ****** **                                    *

D.m. QAEQSAHR
T.c. RSSPAE--
F.o. ASPAAS--
F.c. SSPPHS--
E.d. HSSPAS--
```

Additional file 2: Fig. S9 Amino acid alignment of putative *Ephemera danica* (E.d.) Zen with homologs from four additional hexapod species. Sequence annotations marked in bold, as follows: HD, Homeodomain (blue); C-terminus (purple); linker region is in black and not boldfaced. Species abbreviations are as follows: *Drosophila melanogaster,* D.m.; *Tribolium castaneum*, T.c.; *Frankliniella occidentalis*, F.o.; *Folsomia candida*, F.c.; *Ephemera danica*, E.d. All sequence accession numbers are reported in Additional file 1: Table S1.

```
       Hx                                          HD
     1                                                          60
     |         |         |         |         |         |        |
D.m. DFNWSH---------------IEETLASDCKDSKRTRQTYTRYQTLELEKEFHFNRYITR
T.c. FYPWMK-----------------AHGDSSATGNKRTRQTYTRYQTLELEKEFHFNKYLTR
H.h. IYPWMI-----------------SSDANRPGGTKRTRQTYSRYQTLELEKEFHYNKYLSR
F.c. YPPWLKRGSYGLKNTTSPRSPSSEDNISPSSSSKRTRQTYTRCQTLELEKEFHFNKYLTR
E.d. YYPWMK------------------SYTDSGQRSKRTRQTYTRHQTLELEKEFHFNRYLTR
        *                             ******* * ********** * *  *
                                                C-terminus
              70                                      110
               |         |         |         |         |
D.m. RRRIDIANALSLSERQIKIWFQNRRMKSKKDRTLDSSPEHCGAGYTAMLPPLEA--T
T.c. RRRIEIAESLRLTERQIKIWFQNRRMKAKKDTKFTEQSVTSTFDFLSLHPAETS--M
H.h. KRRIEISNELRLTERQIKIWFQNRRMKLKKEIGRT--------------LGEPSQMV
F.c. RRRLDLAKMLTLSERQIKIWFQNRRMKAKKEVKGH------------VVASDLVQRH
E.d. RRRIEIAHTLSLTERQIKIWFQNRRMKAKKDIKFT--------------PTESA--V
      **      * * ************** **
```

Additional file 2: Fig. S10 Amino acid alignment of putative *Ephemera danica* (E.d.) Ftz with homologs from four additional hexapod species. Sequence annotations marked in bold, as follows: Hx, Hexapeptide motif (green); HD, Homeodomain (blue); C-terminus (purple); linker region is in black and not boldfaced. Species abbreviations are as follows: *Drosophila melanogaster*, D.m.; *Tribolium castaneum*, T.c.; *Halyomorpha halys*, H.h.; *Folsomia candida*, F.c. All sequence accession numbers are reported in Additional file 1: Table S1.

```
                1                                                          60
                |        |         |         |         |         |         |
E.d. Ftz        M----QTESDVTKTPPPSTAYYPWMKSYTDSGQRSKRTRQTYTRHQTLELEKEFHFNRYL
C.d. chimera    ----------------------------------SKRTRQTYTRHQTLELEKEFHYNRYV
E.d. Scr        MVGARKCRADIMAVKPRT----------VNANGETKRQRTSYTRYQTLELEKEFHFNRYL
                                                  :** * :***:**********:***:
                             HD
                         70                                                120
                         |         |         |         |         |         |
E.d. Ftz        TRRRRIEIAHTLSLTERQIKIWFQNRRMKAKKDIKFTPTESAVGSIDLNSRMVPELPSTA
C.d. chimera    TRNRRLEIARTLQLTERQIKI-----------------CSSA------------------
E.d. Scr        TRRRRIEIAHALCLTERQIKIWFQNRRMKWKKEHKMANMHLA--SLQDEAMCYQMGPSMH
                **.**:***::* ********                    *
                        130
                         |
E.d. Ftz        KLVPHFGGL--
C.d. chimera    ------GSTW-
E.d. Scr        RGLYACGGPYG
                      *.
```

Additional file 2: Fig. S11 Alignment of an additional homeodomain sequence within *Cloeon dipterum* (C.d.) Scr, indicating that *C. dipterum* Scr is a chimeric sequence containing a possible Ftz homeodomain. Homeodomain sequences are highlighted in blue. All sequence accession numbers are reported in Additional file 1: Table S1.

```
     Hx
    1                                                          60
    |    |    |    |    |    |    |    |    |    |    |    |
D.m TYKWMQLKRNVPKPQAPK-LPASGIASMHDYQMNGQLDMCRGGGGGGSGVGNGPVGVGGN
T.c TYKWMQVKRNVPKPTVPKIPPAEFPTTS------------------SSS---ALESPNPA
F.o TYKWMQVKRNVPKPAVPK-AAAEALREL--------------GCGGAGSVGVGFAGVGGA
Hex TYKWMQVKRNVPKPA-PK-PSEYYPGSP-------------SMVPVSSPLQVGLPGGGGL
E.d TYKWMQVKRNVPKPA-PK-PSEYYPGSP------------ASMVPVSNSLQGGLPGGGGL
C.d TYKWMQVKRNVPKPA-SK-ATVELFQQHH------Q----STSLPIGPS---AMAGSGPL
    ****** *******   *
             70                                             120
             |         |         |         |         |         |
D.m GSPGIGGVLSVQNSLIMANSAAAAGSAHPNGMGVGLGSGSGL-----SSCSLSSN-TNNS
T.c S------------------------------------RSSCL--GSNTS-SMLSLNCLNT
F.o SLP----------------AGASAG-------GVGVGPALSL--SPSASCPAAAL-LANS
Hex S-P----------------LGAS-----------GLHQGHQLHPGAGGA-AALAA-AAGA
E.d S-P----------------L--------------GLHQNHQL--GVGGA-AALAA-AAGA
C.d G------------------------------------PLSSL--GPVLS-GAAAL-AAGA
                                             *
                               HD
             130                                            180
             |         |         |         |         |         |
D.m GRTNFTNKQLTELEKEFHFNRYLTRARRIEIANTLQLNETQVKIWFQNRRMKQKKRVKEG
T.c GRTNFTNKQLTELEKEFHFNKYLTRARRIEIASALQLNETQVKIWFQNRRMKQKKRMKEG
F.o GRTNFTNKQLTELEKEFHFNKYLTRARRIEIASALQLNETQVKIWFQNRRMKQKKRLKEG
Hex GRTNFTNKQLTELEKEFHFNKYLTRARRIEIASALQLNETQVKIWFQNRRMKQKKRMKEG
E.d GRTNFTNKQLTELEKEFHFNKYLTRARRIEIASALQLNETQVKIWFQNRRMKQKKRMKEG
C.d GRTNFTNKQLTELEKEFHFNRYLTRARRIEIASALQLNETQVKIWFQNRRMKQKKRAKEG
    ******************** ***********  ********************** ***
        C-terminus
                       200
             |         |
D.m LIPADILTQH----STS---
T.c LIPPEPISAS----LST---
F.o LVPPDIGSSSAAGASAGSGG
Hex LIPPEPLQVAGGATSPTS--
E.d LIPPEQLQVVGGATSPTS--
C.d LIPPESVATS----LPSS--
    * *
```

Additional file 2: Fig. S12 Amino acid alignment of putative *H. limbata (Hex)* Lab with homologs from five additional hexapod species. Sequence annotations marked in bold, as follows: Hx, Hexapeptide motif (green); HD, Homeodomain (blue); C-terminus (purple); linker region is in black and not boldfaced. Dashes indicate gaps in the sequence, asterisks label identical residues. Species abbreviations are as follows: *D.m., Drosophila melanogaster*; *T.c.*, *Tribolium castaneum*; *F.o.*, *Frankliniella occidentalis*; *E.d.*, *Ephemera danica*; *C.d.*, *Cloeon dipterum*. All sequence accession numbers are reported in Additional file 1: Table S1.

```
      Hx                                               HD
    1                                                          60
    |         |         |         |         |         |         |
D.m EYPWMKEKKTSRKSSNNNNQGDNSITEFVPENGLPRRLRTAYTNTQLLELEKEFHFNKYL
T.c EYPWMKEKKTTRKSSQ-------------QENGLPRRLRTAYTNTQLLELEKEFHFNKYL
F.o EYPWMKEKKTTRKSSQ-------------QENGLPRRLRTAYTNTQLLELEKEFHFNKYL
O.c -----------------------------MNNGMPRRLRTAYTNTQLLELEKEFHFNKYL
Hex EYPWMKEKKTTRKSNA-------------QENGMPRRLRTAYTNTQLLELEKEFHFNKYL
E.d ---------------------------------MPRRLRTAYTNTQLLELEKEFHFNKYL
C.d EYPWMKEKKTSRKNST-------------PENGLPRRLRTAYTNTQLLELEKEFHFNKYL
                                      **************************
                                              C-terminus

             70                                      110
             |         |         |         |         |
D.m CRPRRIEIAASLDLTERQVKVWFQNRRMKHKRQTLSKT----DDEDNKDSLKGD---
T.c CRPRRIEIAASLDLTERQVKVWFQNRRMKHKRQTLGKQG---DDGDDKDSITSDGGK
F.o CRPRRIEIAASLDLTERQVKVWFQNRRMKHKRQTLSKSG---DDEDSKDAPGRS--K
O.c CRPRRIEIAASLDLSERQVKVWFQNRRMKHKRQTISKSSGGEDGEKSTDGKSSTKGS
Hex CRPRRIEIAASLDLTERQVKVWFQNRRMKHKRQTMGKGD---DEKE-GGGKGKDGKG
E.d CRPRRIEIAASLDLTERQVKVWFQNRRMKHKRQTMGKGEG--DEKEGGGGKSKDGKS
C.d CRPRRIEIAASLDLTERQVKVWFQNRRMKHKRQTVGKGE---DDKESSKEKAK----
    ************** *******************   *     *
```

Additional file 2: Fig. S13 Amino acid alignment of putative *H. limbata (Hex)* Pb with homologs from six additional hexapod species. Sequence annotations marked in bold, as follows: Hx, Hexapeptide motif (green); HD, Homeodomain (blue); C-terminus (purple); linker region is in black and not boldfaced. Dashes indicate gaps in the sequence, asterisks label identical residues. Species abbreviations are as follows: *D.m.*, *Drosophila melanogaster*; *T.c., Tribolium castaneum*; *F.o.*, *Frankliniella occidentalis*; *O.c., Orchesella cincta*; *E.d., Ephemera danica*; *C.d., Cloeon dipterum*. All sequence accession numbers are reported in Additional file 1: Table S1.

```
    SSYF  Bp     Hx                                           HD
    1                                                                60
    |               |         |         |         |         |         |
D.m SSFL -337- IYPWMKKIHVAGV-------------ANGSYQPGMEPKRQRTAYTRHQILELEKEFHYN
T.c SSFL -203- IYPWMRKVHVAGA-------------SNGTFAPGMEPKRQRTAYTRHQILELEKEFHYN
F.o SSYL -150- IYPWMKKIHVAGA-------------SNGGYQPGMEPKRQRTAYTRHQILELEKEFHFN
F.c SSFL -282- IFPWMKKAHIAGK--MPRGGRDSNFVISGS---SLDTKRQRTAYTRHQILELEKEFHYN
Hex ---- -057- IYPWMRKIHVAGVGECPTS------MANGSFQPGMEPKRQRTAYTRHQILELEKEFHFN
E.d FSYL -039- LTTWFFFLFCVFT-------------ANGSFQPGMEPKRQRTAYTRHQILELEKEFHFN
C.d QDAS -160- IYPWMKKIHVAGV-------------AIGQFQPGMEPKRQRTAYTRHQILELEKEFHFN
                                                    ******************** *
                                                   C-terminus

         70                                                120
          |         |         |         |         |         |
D.m RYLTRRRRIEIAHTLVLSERQIKIWFQNRRMKWKKDNKLPNTKNVRKKTVDANGNPTPVA
T.c RYLTRRRRIEIAHTLVLSERQIKIWFQNRRMKWKKDNKLPNTKNVRRKTNPAGVTTTTKG
F.o RYLTRRRRIEIAHTLVLSERQIKIWFQNRRMKWKKDNKLPNTKNVRRKTNPAGVVTTVAG
F.c RYLTRRRRIEIAHSLVLSERQIKIWFQNRRMKYKKDNKLPNTKNVRRKNKQAPVSNHPAS
Hex RYLTRRRRIEIAHSLCLSERQIKIWFQNRRMKWKKDNKLPNTKNVRRKTNPAGVTTT---
E.d RYLTRRRRIEIAHSLCLSERQIKIWFQNRRMKWKKDNKLPNTKNVRRKTNPAGVTTT---
C.d RYLTRRRRIEIAHSLNLSERQIKIWFQNRRMKWKKDNKLPNTKNVRRKN--ANGQPSPP-
    ************* ******************************** *   *
```

Additional file 2: Fig. S14 Amino acid alignment of putative *H. limbata* (*Hex*) Dfd with homologs from six additional hexapod species. Sequence annotations marked in bold, as follows: SSYF motif (red); Bp, count of trimmed base pairs (in grey); Hx, Hexapeptide motif (green); HD, Homeodomain (blue); C-terminus (purple); linker region is in black and not boldfaced. Dashes indicate gaps in the sequence, asterisks label identical residues. Species abbreviations are as follows: *D.m.*, *Drosophila melanogaster*; *T.c., Tribolium castaneum*; *F.o.*, *Frankliniella occidentalis*; *F.c.*, *Folsomia candida*; *E.d.*, *Ephemera danica*; *C.d.*, *Cloeon dipterum*. All sequence accession numbers are reported in Additional file 1: Table S1.

```
    SSYF    Bp      Hx                                    HD
        1                                                      50
        |               |         |         |         |         |
D.m SSYQF -290- IYPWMKRVHLGTSTVNANGETKRQRTSYTRYQTLELEKEFHFNRYLTRRRRIEI
T.c SSYQF -193- IYPWMKRVHLGQSTVNANGETKRQRTSYTRYQTLELEKEFHFNRYLTRRRRIEI
F.o SSYQF -221- IYPWMKRVHLGQSTVNANGETKRQRTSYTRYQTLELEKEFHFNRYLTRRRRIEI
F.c SSYQF -208- IYPWMKRVHLGQS-----------------------------------------
Hex SSYPF -219- IYPWMKRVHLGQSTVNANGETKRQRTSYTRYQTLELEKEFHFNRYLTRRRRIEI
E.d ----- -017- -------------TVNANGETKRQRTSYTRYQTLELEKEFHFNRYLTRRRRIEI
C.d SSYQF -200- IYPWMKRVHLGQSTVNANGETKRQRTSYTRYQTLELEKEFHFNRYLTRRRRIEI

                                       C-terminus
    60                                      100
    |         |         |         |         |
D.m AHALCLTERQIKIWFQNRRMKWKKEHKMASMNIVPYH-------
T.c AHALCLTERQIKIWFQNRRMKWKKEHKMASMNIVPYH-------
F.o AHALCLTERQIKIWFQNRRMKWKKEHKMASMNIVPYYHNMS--M
F.c -----------------------KEIKYS---------------
Hex AHALCLTERQIKIWFQNRRMKWKKEHKMANMHLASLQDEAMCYQ
E.d AHALCLTERQIKIWFQNRRMKWKKEHKMANMHLASLQDEAMCYQ
C.d AHALCLTERQIKIWFQNRRMKWKKEHKMANMHLASLQDEAMCYQ
                           ** *
```

Additional file 2: Fig. S15 Amino acid alignment of putative *H. limbata* (*Hex*) Scr with homologs from six additional hexapod species. Sequence annotations marked in bold, as follows: SSYF motif (red); Bp, count of trimmed base pairs (grey); Hx, Hexapeptide motif (green); HD, Homeodomain (blue); C-terminus (purple); linker region is in black and not boldfaced. Dashes indicate gaps in the sequence, asterisks indicate identical residues. Species abbreviations are as follows: *D.m.*, *Drosophila melanogaster*; *T.c.*, *Tribolium castaneum*; *F.o.*, *Frankliniella occidentalis*; *F.c.*, *Folsomia candida*; *E.d.*, *Ephemera danica*; *C.d.*, *Cloeon dipterum*. All sequence accession numbers are reported in Additional file 1: Table S1.

```
    SSYF  Bp     Hx                            HD
    1                                                       50
    |               |         |         |         |         |
D.m TSYF -267- LYPWMRSQFGKCQERKRGRQTYTRYQTLELEKEFHFNRYLTRRRRIEIAHALCLT
T.c SSYF -228- LYPWMRSQF----ERKRGRQTYTRYQTLELEKEFHFNRYLTRRRRIEIAHALCLT
F.o SSYF -268- LYPWMRSQF----ERKRGRQTYTRYQTLELEKEFHFNRYLTRRRRIEIAHALCLT
O.c TSYF -278- LYPWMRSQF----EIRD-----TAYDNVNV-------------------------
Hex SSYF -230- LYPWMRSQF----ERKRGRQTYTRYQTLELEKEFHFNRYLTRRRRIEIAHALCLT
E.d SSYF -232- LYPWMRSQF----ERKRGRQTYTRYQTLELEKEFHFNRYLTRRRRIEIAHALCLT
C.d SSYF -194- LYPWMRSQF----ERKRGRQTYTRYQTLELEKEFHFNRYLTRRRRIEIAHALCLT
     ***       *********    *        * *
                                 C-terminus

    60                                      100
    |         |         |         |         |
D.m ERQIKIWFQNRRMKWKKENKTKGEPGSGGEGD---EITPPNSP--Q
T.c ERQIKIWFQNRRMKWKKENKTKGEGGSEGGGD---DISPQGSP--Q
F.o ERQIKIWFQNRRMKWKKENKTKGEPGS-GDGDGSGELSPQTSP--Q
O.c ------------------------------------------PIKM
Hex ERQIKIWFQNRRMKWKKENKTKTESGS-GDGM---QGSDPGSPP-Q
E.d ERQIKIWFQNRRMKWKKENKTRTESGS-GDGL---QGSDAGSPPPQ
C.d ERQIKIWFQNRRMKWKKENKSKSDSGSQHDGM---DATPPDSP--Q
```

Additional file 2: Fig. S16 Amino acid alignment of putative *H. limbata* (*Hex*) Antp with homologs from six additional hexapod species. Sequence annotations marked in bold, as follows: SSYF motif (red); Bp, count of trimmed base pairs (grey); Hx, Hexapeptide motif (green); HD, Homeodomain (blue); C-terminus (purple); linker region is in black and not boldfaced. Dashes indicate gaps in the sequence, asterisks label identical residues. Species abbreviations are as follows: *D.m.*, *Drosophila melanogaster*; *T.c.*, *Tribolium castaneum*; *F.o.*, *Frankliniella occidentalis*; *O.c.*, *Orchesella cincta*; *E.d.*, *Ephemera danica*; *C.d.*, *Cloeon dipterum*. All sequence accession numbers are reported in Additional file 1: Table S1.

```
    SSYF  Bp     Hx
    1                                                          60
    |        |         |         |         |         |         |
D.m NSYF -233- FYPWMAIAGECPEDPTKSKIRSDLTQYGGISTDMGKRYSESLAGSLLPDWLGTNGL
T.c NSYF -199- FYPWMAIADSMT--------------------------------------FGANGL
T.p NSYF -206- FYPWMAIAGGC---------------------------------------FGANGL
O.c NSFY -177- FYPWMAIAGFM---------------------------------------TGANGL
Hex NSYF -207- FYPWMAIA-------------------------------------------GANGL
E.d E--- -001- ------VAHG--------------------------------------DEQGANGL
C.d NSYF -180- FYPWMAIAGT-----------------------------------------GANGL
                  *                                           *****
                            HD
             70                                             120
             |         |         |         |         |         |
D.m RRRGRQTYTRYQTLELEKEFHTNHYLTRRRRIEMAHALCLTERQIKIWFQNRRMKLKKEIQAI
T.c RRRGRQTYTRYQTLELEKEFHTNHYLTRRRRIEMAHALCLTERQIKIWFQNRRMKLKKEIQAI
T.p RRRGRQTYTRYQTLELEKEFHTNHYLTRRRRIEMAHALCLTERQIKIWFQNRRMKLKKEIQAI
O.c RRRGRQTYTRYQTLELEKEFHTNHYLTRRRRIEMAHALCLTERQIKIWFQNRRMKLKKEIQAI
Hex RRRGRQTYTRYQTLELEKEFHTNHYLTRRRRIEMAHALCLTERQIKIWFQNRRMKLKKEIQAI
E.d RRRGRQTYTRYQTLELEKEFHTNHYLTRRRRIEMAHALCLTERQIKIWFQNRRMKLKKEIQAI
C.d RRRGRQTYTRYQTLELEKEFHTNHYLTRRRRIEMAHALCLTERQIKIWFQNRRMKLKKEIQAI
    ***************************************************************
     UbdA   QAQA  Poly-A
                  140
          |         |
D.m KELNEQEKQAQAQKAAAAAA
T.c KELNEQEKQAQAQKAAAAAA
T.p KELNEQEKQAQAQKAAAAAA
O.c KELNEQEKQAQAAKVVLHGV
Hex KELNEQEKQAQAQKAAAAAA
E.d KELNEQEKQAQAQKAAAAAA
C.d KELNEQEKQAQAQKAAAAAA
    **************
```

Additional file 2: Fig. S17 Amino acid alignment of putative *H. limbata* (*Hex*) Ubx with homologs from six additional hexapod species. Sequence annotations marked in bold, as follows: SSYF motif (red); Bp, count of trimmed base pairs (grey); Hx, Hexapeptide motif (green); HD, Homeodomain (blue); UbdA motif (orange); QAQA region (yellow); Poly-A region (grey); linker region is in black and not boldfaced. Dashes indicate gaps in the sequence, asterisks label identical residues. Species abbreviations are as follows: *D.m.*, *Drosophila melanogaster*; *T.c.*, *Tribolium castaneum*; *T.p.*, *Thrips palmi*; *O.c.*, *Orchesella cincta*; *E.d.*, *Ephemera danica*; *C.d.*, *Cloeon dipterum*. All sequence accession numbers are reported in Additional file 1: Table S1.

```
    SSYF  Bp     Hx                      TDWM   PFER
    1                                                                 60
    |               |         |         |         |         |         |
D.m ---- -103- RYPWMTL------------------TDWMGSPF--ERVVCGD---------------
T.c SSKF -185- RYPWMSI------------------TDWM-SPF--DRVVC-----------------
F.o TSKF -197- RYPWMSI------------------TDWM-SPF--DRVVCGGPTARQTPGLADIYGD
O.c NSKF -148- RYPWMAIPGSDAHRNMDSATRRSFEMEQFYSPYLLEKQHRAD------------LDC
Hex TSKF -152- RYPWMSI------------------TDWM-SPF--DRVVCD--------------MS
E.d T--- -025- -------------------------------------------------------QS
C.d TSKF -145- RYPWMSI------------------TDWM-SPF--DRVVCGP-------------YD

                                       HD
            70                                                120
            |         |         |         |         |         |
D.m -FNGPNGCPRRRGRQTYTRFQTLELEKEFHFNHYLTRRRRIEIAHALCLTERQIKIWFQNR
T.c ---GPNGCPRRRGRQTYTRFQTLELEKEFHFNHYLTRRRRIEIAHALCLTERQIKIWFQNR
F.o FIRGPNGCPRRRGRQTYTRFQTLELEKEFHFNHYLTRRRRIEIAHALCLTERQIKIWFQNR
O.c WTTGPNGCPRRRGRQTYTRFQTLELEKEFHFNHYLTRRRRIEIAHALCLTERQIKIWFQNR
Hex FVTGPNGCPRRRGRQTYTRFQTLELEKEFHFNHYLTRRRRIEIAHALCLTERQIKIWFQNR
E.d MEDGPNGCPRRRGRQTYTRFQTLELEKEFHFNHYLTRRRRIEIAHALCLTERQIKIWFQNR
C.d GLGSPNGCPRRRGRQTYTRFQTLELEKEFHFNHYLTRRRRIEIAHALCLTERQIKIWFQNR
        *********************************************************
               UbdA          C-terminus
                                        160
           |         |         |         |
D.m RMKLKKELRAVKEINEQARRDREEQEKMKAQETMKSAQQNK
T.c RMKLKKELRAVKEINEQARREREEQERHKQQQQEK------
F.o RMKLKKELRAVKEINEQARREREEQERLKQQQQEK------
O.c RMKLKKELRAVKEINEQARREREEHDKHKPRDSSS------
Hex RMKLKKELRAVKEINEQARREREEQERLKQQQQQQ------
E.d RMKLKKELRAVKEINEQARRERDEQERLKQQQQQQ------
C.d RMKLKKELRAVKEINEQARREREEQERIKSQQQQQ------
    ******************** * *    *
```

Additional file 2: Fig. S18 Amino acid alignment of putative *H. limbata* Abd-A with homologs from six additional hexapod species. Sequence annotations marked in bold, as follows: SSYF motif (red); Bp, count of trimmed base pairs (grey); Hx, Hexapeptide motif (green); TDWM motif (yellow); PFER motif (light blue); HD, Homeodomain (blue); UbdA motif (orange); C-terminus (purple); linker region is in black and not boldfaced. Dashes indicate gaps in the sequence, asterisks label identical residues. Species abbreviations are as follows: *D.m.*, *Drosophila melanogaster*; *T.c.*, *Tribolium castaneum*; *F.o.*, *Frankliniella occidentalis*; *O.c.*, *Orchesella cincta*; *E.d.*, *Ephemera danica*; *C.d.*, *Cloeon dipterum*. All sequence accession numbers are reported in Additional file 1: Table S1.

```
      Hx                                 HD
    1                                                 50
    |         |         |         |         |         |
D.m LHEWTGQVSVRKKRKPYSKFQTLELEKEFLFNAYVSKQKRWELARNLQLTERQVKIWF
T.c -LEWTGQVTVRKKRKPYSKFQTLELEKEFLFNAYVSKQKRWELARNLNLTERQVKIWF
T.p -LEWTGQVTVRKKRKPYSKFQTLELEKEFLFNAYVSKQKRWELARNLNLTERQVKIWF
O.c -LEWTGNVTVRKKRKPYSKFQTLELEKEFLFNAYVSKQKRWELARNLNLTERQVKIWF
Hex -LEWTGNVTVRKKRKPYSKFQTLELEKEFLFNAYVSKQKRWELARNLNLTERQVKIWF
E.d -LEWTGNVTVRKKRKPYSKFQTLELEKEFLFNAYVSKQKRWELARNLNLTERQVKIWF
C.d -LEWAGNVTVRKKRKPYSKFQTLELEKEFLFNAYVSKQKRWELARNLNLTERQVKIWF
      ** * * ************************************** **********
                     C-terminus
     60                            90
     |         |         |         |
D.m QNRRMKNKKNSQRQANQQNNNNNSS---SNHNHAQ
T.c QNRRMKNKKNSQRQAAQQQNNNNAATNNQNHHH-H
T.p QNRRMKNKKNSQRQAAQQNNNSSSASNNNHHHH--
O.c QNRRMKNKKNSQRQAAQQASQSQA---QDHHGH--
Hex QNRRMKNKKNSQRQSAQQANSSTGA--GGHHGH--
E.d QNRRMKNKKNSQRQSAQQTSSS--G--GGHHGH--
C.d QNRRMKNKKNSQRQAAQQNNSS------NHHGH--
    **************  **            * *
```

Additional file 2: Fig. S19 Amino acid alignment of putative *H. limbata* (*Hex*) Abd-B with homologs from six additional hexapod species. Sequence annotations marked in bold, as follows: Hx Region, Hexapeptide region (green); HD, Homeodomain (blue); C-terminus (purple). Dashes indicate gaps in the sequence, asterisks label identical residues. Species abbreviations are as follows: *D.m.*, *Drosophila melanogaster*; *T.c.*, *Tribolium castaneum*; *T.p.*, *Thrips palmi*; *O.c.*, *Orchesella cincta*; *E.d.*, *Ephemera danica*; *C.d.*, *Cloeon dipterum*. All sequence accession numbers are reported in Additional file 1: Table S1.
